# Probing Interfacial Nanostructures of Electrochemical Energy Storage Systems by In-Situ Transmission Electron Microscopy

**DOI:** 10.1007/s40820-025-01720-5

**Published:** 2025-04-30

**Authors:** Guisheng Liang, Chang Zhang, Liting Yang, Yihao Liu, Minmin Liu, Xuhui Xiong, Chendi Yang, Xiaowei Lv, Wenbin You, Ke Pei, Chuan-Jian Zhong, Han-Wen Cheng, Renchao Che

**Affiliations:** 1 Laboratory of Advanced Materials, Shanghai Key Lab of Molecular Catalysis and Innovative Materials, Department of Materials Science, Academy for Engineering and Technology, Fudan University, Shanghai, 200438 People’s Republic of China; 2 Department of Chemistry, State University of New York at Binghamton, Binghamton, NY 13902 USA

**Keywords:** In-situ transmission electron microscopy, Electrochemical energy storage, Interfacial nanostructures, Batteries, Electrodes, Nanomaterials

## Abstract

The ability to control the electrode interfaces in an electrochemical energy storage system is essential for achieving the desired electrochemical performance. However, achieving this ability requires an in-depth understanding of the detailed interfacial nanostructures of the electrode under electrochemical operating conditions. In-situ transmission electron microscopy (TEM) is one of the most powerful techniques for revealing electrochemical energy storage mechanisms with high spatiotemporal resolution and high sensitivity in complex electrochemical environments. These attributes play a unique role in understanding how ion transport inside electrode nanomaterials and across interfaces under the dynamic conditions within working batteries. This review aims to gain an in-depth insight into the latest developments of in-situ TEM imaging techniques for probing the interfacial nanostructures of electrochemical energy storage systems, including atomic-scale structural imaging, strain field imaging, electron holography, and integrated differential phase contrast imaging. Significant examples will be described to highlight the fundamental understanding of atomic-scale and nanoscale mechanisms from employing state-of-the-art imaging techniques to visualize structural evolution, ionic valence state changes, and strain mapping, ion transport dynamics. The review concludes by providing a perspective discussion of future directions of the development and application of in-situ TEM techniques in the field of electrochemical energy storage systems.

## Highlights

- An in-depth look into the latest developments of in-situ transmission electron microscopy (TEM) imaging techniques for probing the interfacial nanostructures of electrochemical energy storage systems.
- Selected examples to highlight the fundamental understanding of atomic-scale and nanoscale mechanisms by employing some of the state-of-the-art imaging techniques to visualize the interfacial nanostructural evolution.
- The challenges and future directions of the development and application of in-situ TEM techniques in the cutting-edge areas of electrochemical energy storage research are discussed.

## Graphical abstract

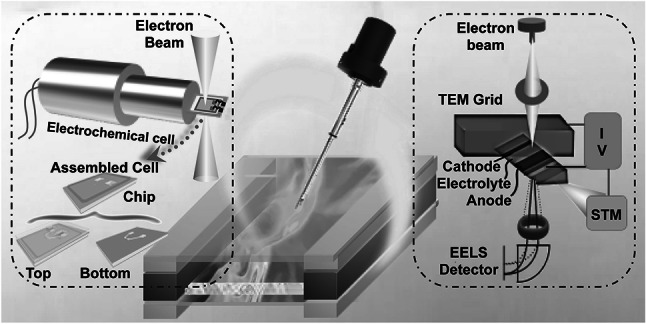

## Introduction

The development of lithium-ion batteries (LIBs), especially the rechargeable ones, has changed the world since the pioneering work by Nobel Laureates Whittingham, Goodenough, and Yoshino about half a century ago [1]. LIBs are today an essential part of our daily life, driving the development of various electrochemical energy storage systems for electrifying the world by shifting the global energy consumption away from fossil fuels and toward electricity produced from renewable sources [2–7]. Indeed, electrochemical energy storage is becoming part of the global drive seeking alternatives to fossil fuels, including various renewable and clean sources (solar energy, wind energy, biomass energy, etc.). The need of efficient conversion and storage for these energy sources constitutes a major driving force for innovations in energy conversion and storage systems, such as lithium-ion or metal-air batteries for solar energy storage and hydrogen production through electrolysis of water, fuel cells for converting hydrogen into electricity [8]. The water-splitting hydrogen production from renewable solar or wind sources and the fuel cell conversion of hydrogen to electricity has become a sustainable power package that address many of the challenges of energy and environmental sustainability [8–12]. Electrochemical energy storage devices or systems play a crucial role in the development of clean and sustainable energy in modern society. Applications of such devices span across aerospace, artificial intelligence, electric vehicles, and many other fields [13–18]. Among various electrochemical energy storage solutions, rechargeable secondary batteries such as Li/Na/K/Zn/Mg-ion batteries [19–27], metal-air batteries [28–31], and all-solid-state batteries [32], are widely adopted due to their efficient energy storage capabilities. Typically, the energy storage density depends on the structure of the electrode materials and their electrochemical properties. This dependence reflects the correlation of the dynamic evolution of the electrode/electrolyte interphase layer and the ionic/electronic transport behavior with the cycling stability and power density of the batteries. Under practical operating conditions, it is difficult to examine the reactions occurring within a sealed battery using conventional characterization methods. Therefore, the precise determination of the fundamental mechanism for the electrochemical reactions and structural evolution under dynamic operating conditions has been a long-standing subject of debate, especially in area concerning the atomic-scale structural evolution of electrode materials, the formation of solid electrolyte interphase, and ion transport kinetics [33–35]. Understanding the correlation between materials structure, electrochemical processes, and electronic properties during repeated electrochemical cycling holds the key to developing high-performance rechargeable secondary batteries.

To probe reaction kinetics and interfacial structure evolution in electrochemical energy storage devices during cycling, various in-situ spectroscopic or microscopic techniques have been developed, including optical microscopy, scanning electron microscopy (SEM) [36], X-ray diffraction (XRD) [37–40], nuclear magnetic resonance (NMR) spectroscopy [41], transmission X-ray microscopy (TXM) [42], and Raman spectroscopy [43]. These techniques allowed characterizations of electrodes, electrolytes, and their interfaces. For example, in-situ SEM is used to track the morphological evolution of electrode surfaces, uncovering the formation mechanism of lithium dendrites [44]. In-situ XRD is applied to examine phase transformations [40, 45], revealing the relationship between phase structure and electrochemical reaction. When comparing these in-situ techniques, in-situ TEM has distinct advantages, including i) the capability to track changes in materials, providing real-time observation, and ii) the highest spatial resolution to determine atomic-scale details of phase structure [46–53], ion transport, and chemical valence states. These are important for understanding the detailed electrochemical reaction mechanisms. Other techniques such as XPS, XRD, Raman, and FTIR, on the other hand, can only provide microscopic average information due to the low spatial coherence of X-ray, neutron, and infrared ray, etc. Moreover, with the aid of diverse sample rods and accessories, in-situ TEM can meet various experimental needs by providing the most comprehensive capabilities, including characterizing atomic-scale structure, elemental types, valence states, ratios of different elements, and coordination environments, while other techniques only have a subset of these capabilities. In 2010, the development of in-situ electron transmission electron microscopy (TEM) demonstrated the viability of visualizing the electrochemical reaction process [54]. To date, in-situ TEM has become a powerful technique to reveal the correlation between electrode microstructure and electrochemical performance, which benefits from high spatial/temporal resolution, direct visualization capability and superior sensitivity to electrical structure [55, 56]. Particularly, the introduction of aberration correctors enables the investigation of nanocrystals and nanostructures at atomic resolution. Thus, in-TEM techniques have allowed realization of micro- to macro-length characterizations of atomic structures during battery operation. Indeed, in-situ TEM techniques have been employed for studying intercalation in 2D materials [57], visualizing the formation of LiF nanosheets at the cathode-electrolyte interface in liquid-electrolyte LIBs [58], and visualizing rechargeable battery reactions [59]. Significant progress has been made in design of in-situ cells [38, 39] and the fabrication of liquid cells for in-situ TEM observation of electrochemical processes [60].

While significant progress has been made in advancing various electrochemical energy storage systems and in applying TEM and in-situ TEM techniques for studying the structures of the electrode materials in some of the storage systems, the understanding of the how the nanoscale structures of the electrochemical interfaces correlate with the electrochemical performance remains elusive. In this review, we highlight some of the latest advancements in developing and applying in-situ TEM techniques for the interfacial nanostructures, including atomic-scale structural imaging, electron energy-loss spectral imaging, strain field mapping, electron holography, and state-of-the-art electron microscopy instrumentation. These cutting-edge techniques allow for the visualization of atomic-structural changes, ionic valence state transitions, strain mapping, ion transport dynamics, and the evolution of locally polarized electric fields, offering deep insights into the atomic-scale and nanoscale mechanisms. Built upon our understanding of some of the challenging issues and new approaches in lithium batteries through characterizations using TEM and other techniques [37–40, 61–72], and the influence of the electron beam in in-situ TEM observations [46, 73, 74], this review will also discuss key challenges, current solutions, and future directions in the development and applications of in-situ TEM techniques for probing interfacial structures and mechanisms in various electrochemical energy storage systems.

## Use of In-Situ TEM in Electrochemical Energy Storage Systems

**Fig. 1:**
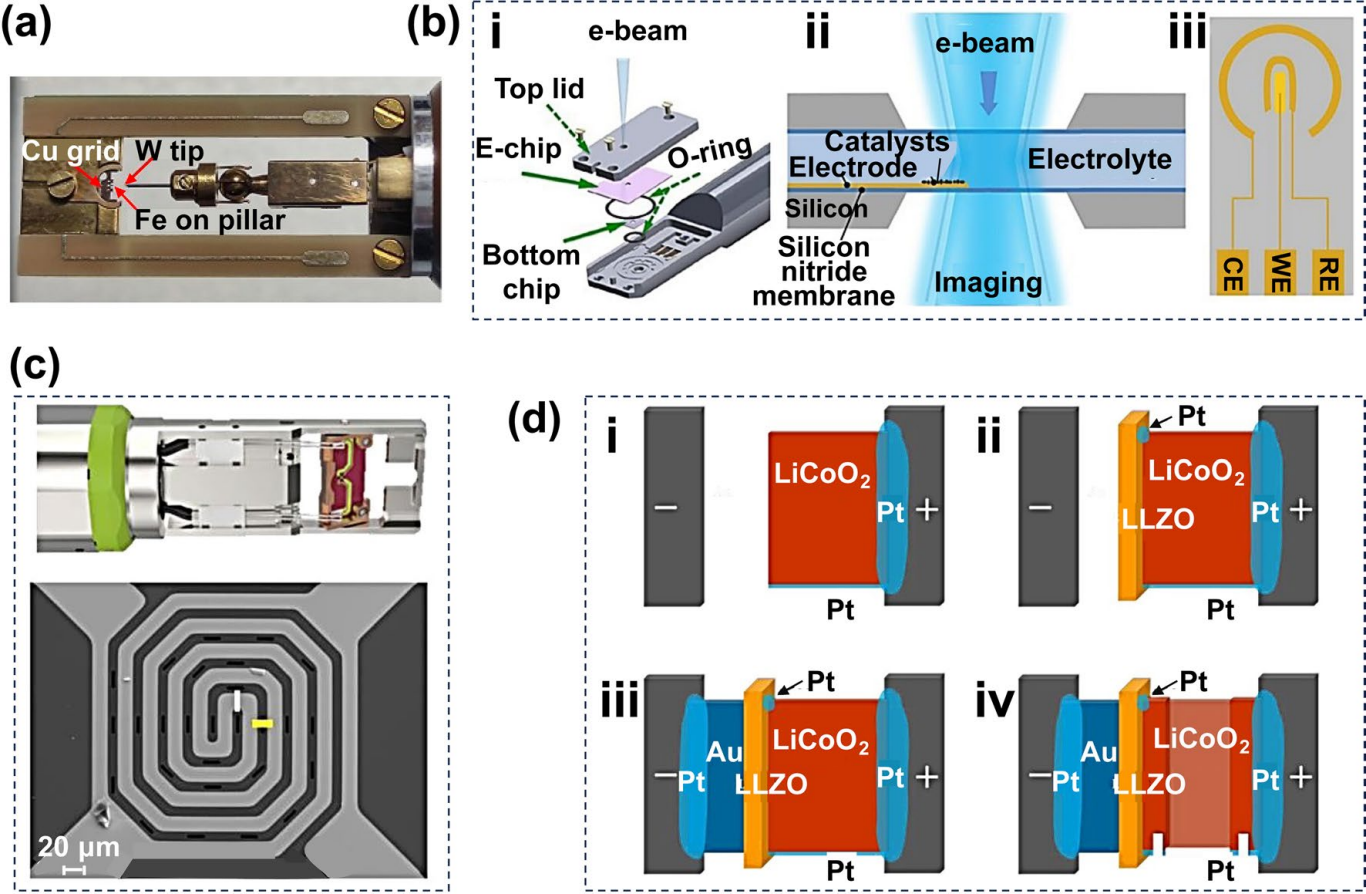
**a** Schematic of the probe-type in-situ TEM holder. Reproduced with permission from Ref. [75]. Copyright 2023, Elsevier. **b** Schematic example of a liquid in-situ TEM holder. (i) The internal structure of the holder. (ii) An in-situ electrochemical liquid cell placed in the TEM optical path, and (iii) an electrochemical microchip with electrodes: counter (CE), working (WE), and reference electrode (RE). Reproduced with permission from Ref. [76]. Copyright 2022, OAE Publishing Inc. **c** Top: Schematic of the chip-based in-situ TEM holder; bottom: SEM image of the chip. **d** The process of assembling solid-state batteries using FIB technology. Reproduced with permission from Ref. [85]. Copyright 2017, American Chemical Society

In-situ TEM observation techniques offer unique insights into the electrochemical processes and reaction mechanisms of rechargeable battery materials. The TEM holders are indispensable components for in-situ experiments. To date, various types of in-situ TEM holders have been designed and manufactured to meet different requirements. Specialized in-situ TEM holders are used to create a miniature electrochemical cell that fits inside the TEM chamber. As shown in Fig. 1, the in-situ TEM holders can be categorized into the following three types: probe-type in-situ TEM holder [75], liquid in-situ TEM holder [76], and chip-based in-situ TEM holder [85].

**Fig. 2:**
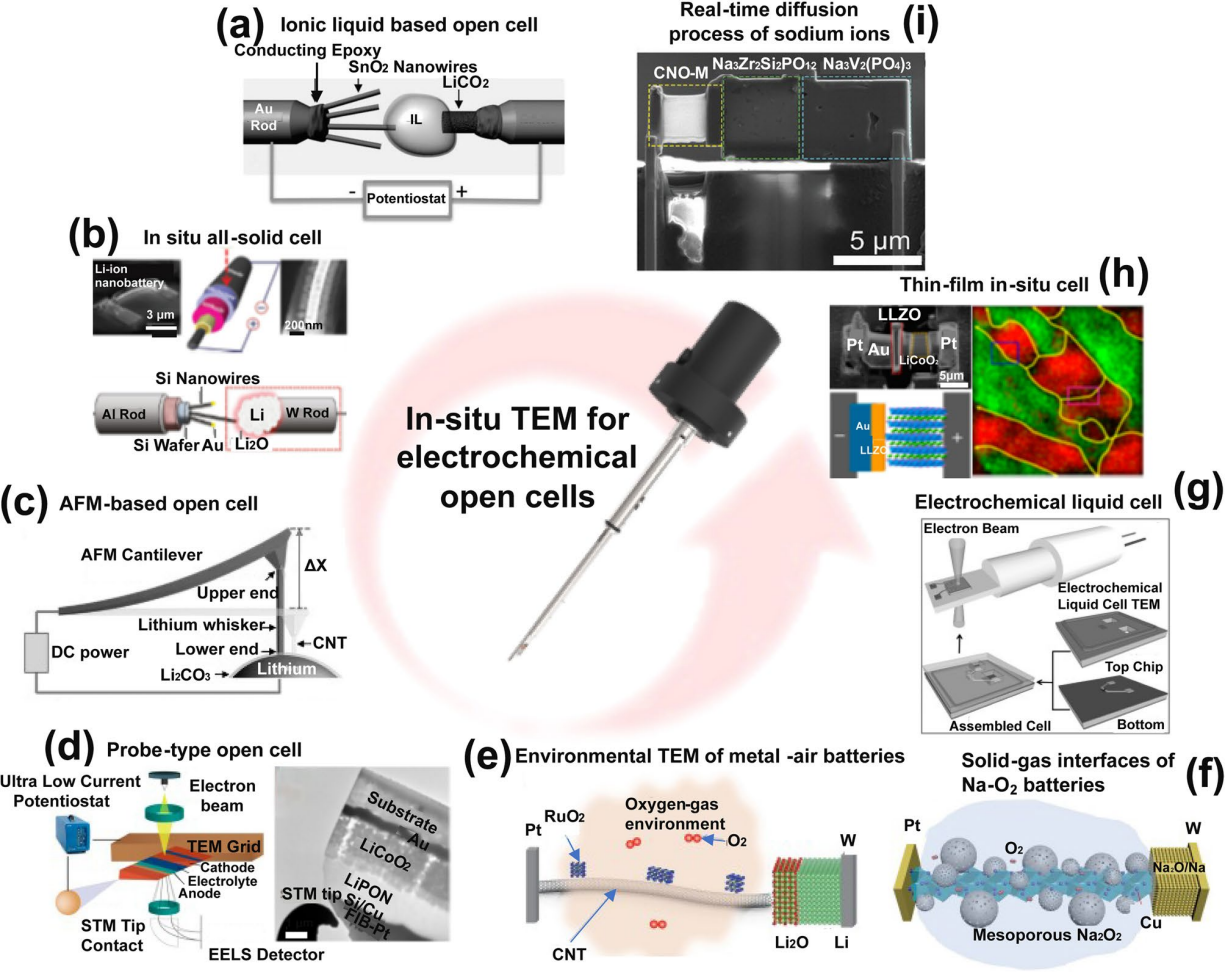
Examples demonstrating the utilization of in-situ TEM in electrochemical open cells. **a** Probe-type electrochemical open cell with ionic liquid electrolyte. Reproduced with permission [54], Copyright 2010, American Association for the Advancement of Science. **b** Probe-type solid-state battery. Reproduced with permission [78, 79], Copyright 2011, American Chemical Society; Copyright 2012, American Chemical Society. **c** AFM-based open cell. Reproduced with permission [80], Copyright 2020, Springer Nature. **d** Probe-type thin-film cell. Reproduced with permission [81], Copyright 2016, American Chemical Society. **e** Probe-type Li-air batteries cell for in-situ environmental TEM observation. Reproduced with permission [82], Copyright 2017, Springer Nature. **f** Probe-type Na-air batteries cell for in-situ environmental TEM observation. Reproduced with permission [83], Copyright 2020, American Chemical Society. **g** Example of an electrochemical liquid cell. Reproduced with permission [84], Copyright 2014, American Chemical Society. **h** All solid-state open cell. Reproduced with permission [85], Copyright 2017, American Chemical Society. **i** In-situ TEM for real-time diffusion process of sodium ions. Reproduced with permission [67], Copyright 2022, Wiley

Probe-type in-situ battery technology was specifically developed to observe the morphology and structural evolution of nanoscale electrode materials in various atmospheric environments. Three types of open in-situ batteries can be assembled on the probe-type in-situ TEM holder, namely ionic liquid probe-type in-situ batteries, all solid probe-type in-situ batteries, and thin-film in-situ batteries. As illustrated in Fig. 2a, the construction of in-situ battery with an ionic liquid probe is primarily composed of two key parts: the anode and the cathode materials. These two parts are fixed at the sharp end of a metal rod coated with conductive organic substances. By introducing a certain amount of electrolyte onto the electrode surfaces, the assembly of the in-situ battery simulates the configuration environment of an actual battery [54]. In this type of in-situ batteries, Li metal acts as the anode where a Li_2_O layer can be formed by the surface oxidation. The surface oxidation of the Li metal results in the formation of a solid electrolyte layer of Li_2_O [77]. Subsequently, by moving the probe, a contact is established between the electrode material and the Li_2_O solid electrolyte, thereby constructing a simple type of solid-state battery (Fig. 2b) [78]. To investigate the growth mechanism and mechanical properties of lithium dendrites, Zhang and coworkers made innovative improvements to the traditional probe-type in-situ configuration (Fig. 2c) [80]. By replacing the conventional metal rod current collector with an atomic force microscope (AFM) probe, real-time stress transmission was realized. Unlike probe-type in-situ batteries, probe-type thin-film in-situ batteries require the use of focused ion beam (FIB) for mid-assembly. By employing FIB technology for precise processing, the thickness of thin-film batteries can be controlled to less than 100 nm. This operation significantly facilitates the efficient acquisition of electronic structure information of electrode materials through electron energy loss spectroscopy (EELS) and electron holography techniques (Fig. 2d) [81]. With the advancement of environmental transmission electron microscopy (ETEM) technology, it has become feasible to observe chemical reaction processes at solid–gas interfaces of metal-air batteries (Fig. 2e, f) [82, 83].

With the liquid in-situ TEM holder, high-resolution TEM maps can be obtained for the battery materials in a liquid environment, facilitating the study of chemical reactions, nanoparticle growth, and electrochemical reactions. For example, Zeng et al. [84] employed such a liquid electrochemical in-situ TEM setup for real-time observation of the formation and growth of solid electrolyte interphase (SEI) and lithium dendrites (Fig. 2g). This configuration allows for simulating the electrochemical reaction environment close to actual operating conditions.

Unlike the other two types of sample holders, experiments based on chip-in-situ TEM holders rely on FIB technology to assemble all-solid-state batteries (Fig. 2h) [85]. Therefore, various combinations of electrode materials and solid-state electrolytes can be implemented on this type of in-situ TEM holders. Additionally, chip-based in-situ TEM holders can be employed to investigate the electrochemical reaction mechanisms of solid-state batteries at different temperatures, which is not available for the other two types of sample holders. In general, the chip-based in-situ TEM holders are primarily used to study electrochemical reaction processes inside all-solid-state batteries. The reaction processes involve structural changes at the electrode/electrolyte interface during charge/discharge cycles, the migration dynamics of ions, and charge transfer at the electrode–electrolyte interface. With FIB technology, the thickness of the viewing area of all-solid-state batteries can be reduced to less than 100 nm, enabling high-resolution atomic-level imaging in STEM mode. In 2022, Liang et al. attempted to use in-situ TEM to observe the real-time diffusion of sodium ions within the anode lattice of thin-film in-situ batteries (Fig. 2i) [67]. However, due to limited ability to adjust the crystal orientation, only the electrochemical reaction front was observed. The reactions and degradation mechanisms of various anode or cathode materials, as well as electrolytes within rechargeable batteries have been monitored and observed in real time using the in-situ open-cell TEM technology, which has significant advantages in studying the structural/chemical evolution of battery materials during cycling. This method provides valuable insights into the microscopic behavior of the battery materials under operating conditions, which calls for further visualization of the detailed interfacial nanostructural evolution.

## In-Situ Visualization of Nanostructural Evolution

### Morphological and Nanostructural Evolution of Electrode Materials

**Fig. 3:**
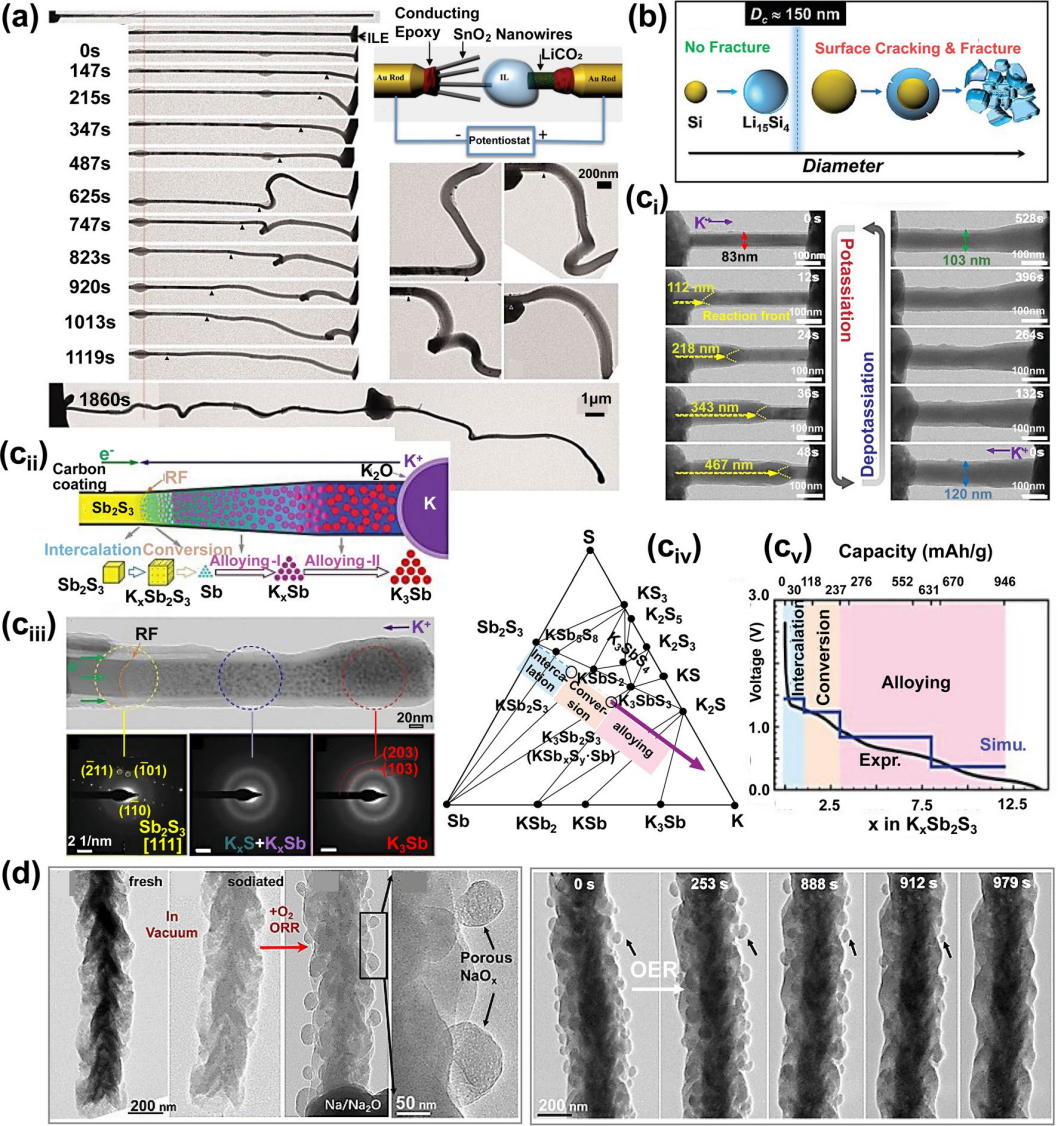
Morphological and nanostructural evolution. **a** Nanostructural evolution of a SnO_2_ nanowire anode during lithiation. Reproduced with permission [54], Copyright 2010, American Association for the Advancement of Science. **b** Diagram of stress-induced silicon nanoparticle fracture. Reproduced with permission [90], Copyright 2012, American Chemical Society. **c** Investigation of electrochemical reaction by combining the in-situ TEM and DFT calculation. (i) In-situ visualization of the morphological evolution of a Sb_2_S_3_@Carbon nanowire during cycling. (ii-iii) Scheme (ii) and TEM image (iii) show the growth and phase evolution of rocket-launching-like NP during potassiation process. (iv) Phase diagram of K-Sb-S calculated by DFT. (v) Comparison of the voltage curve calculated (Simu.) using the intermediate phase with the experimental curve (Expr.). Reproduced with permission [91], Copyright 2020, Wiley. **d** In-situ TEM observation of sodiated CuS nanowire during ORR (left) and OER process (right). Reproduced with permission [83], Copyright 2020, American Chemical Society

During the electrochemical reaction, the electrode materials of Li/Na/K-ion batteries (i.e., LIBs/NIBs/KIBs) inevitably undergo phase transitions or unit-cell volume changes. This electrochemically driven structural evolution leads to large morphological changes in the electrodes, limiting the reversible capacity and cycle lifetime of batteries [86–88]. To comprehensively understand this invalidation mechanism, in-situ TEM has been widely employed to record the electrochemically driven structural and morphological evolutions of electrode materials. For conversion-type and alloy-type electrode materials, the volumetric expansion caused by electrochemical insertion is considered as the primary factor leading to the decline in electrochemical capacity. Reducing the particle size of electrode materials effectively mitigates the capacity decay caused by dimensional changes. To study this enhanced mechanism, Huang et al. directly observed the lithiation process of SnO_2_ nanowire anode in a nanoscale battery of LiCoO_2_||liquid–based electrolyte||SnO_2_ nanowire using TEM (Fig. 3a) [54]. During charging, the lithiation reaction initiates at a single point along the SnO_2_ nanowires and gradually extends along its direction, leading to the expansion, elongation, and bending of the nanowires. Due to the faster lithiation rate on the surface of nanowires, the external expansion of the nanowires exceeds the internal expansion, causing the nanowires to bend. SnO_2_ nanowires maintain structural integrity by bending to accommodate volume expansion, thereby enhancing cycling stability of the battery.

During lithiation, silicon nanoparticles undergo significant volumetric expansion (~ 300%), which results in the generation of substantial internal stress within the particles [89]. In-situ TEM observations reveal that when the particle size is relatively large, the distribution of this stress within the particle is uneven, particularly between the surface and core of the particle. This uneven stress distribution causes the surface layer to experience hoop tension, thereby promoting the formation and propagation of cracks. However, silicon nanoparticles smaller than the critical size (~ 150 nm) do not fracture during first lithiation, whereas the larger particles initially form surface cracks and then fracture due to lithiation-induced swelling (Fig. 3b) [90].

Unlike alloy-type electrode materials, conversion-type materials undergo irreversible phase transformation in the first cycle, leading to changes in the composition and/or structure of the electrodes. As shown in Fig. 3c, the potassiation process of Sb_2_S_3_@Carbon nanowires was recorded via in-situ TEM [91]. During potassiation, the volume of the nanowires gradually expanded, which was accompanied by the production of a significant amount of Sb nanoparticles. Based on the in-situ TEM observation, DFT calculation was performed, revealing multiple reaction pathways of the nanowires. Besides, three distinct reaction types were observed: intercalation (Sb_2_S_3_ → K_*x*_Sb_2_S_3_), conversion (K_*x*_Sb_2_S_3_ → K_*x*_S + Sb), and alloying (K_*x*_S + Sb → K_2_S + K_3_Sb_2_). In conversion stage, accompanied by the generation of Sb nanoparticles and K_*x*_S, the nanowires underwent significant volumetric expansion. The K_*x*_S acted as an electrolyte to transport K-ions, facilitating further alloy evolution of Sb nanoparticles, which led to the further expansion of nanowires. Benefit from this reaction mechanism, conversion-typed electrode materials are widely used as cathode of Li/Na/K–O_2_ batteries. For instance, Han and coworkers employed CuS nanowires as the cathode material for Na–O_2_ batteries and tracked the reversible oxygen reduction reaction (ORR) and oxygen evolution reaction (OER) behaviors of CuS nanowires using ETEM (Fig. 3d) [83]. The CuS nanowires were converted into Cu nanoparticles and Na_*x*_S in the sodiation process. Under the oxygen atmosphere, the Na_*x*_S was transformed into Na_2_O_2_ porous spheres in ORR process, which was evenly distributed on the nanowires surface. In subsequent OER, the formed Na_2_O_2_ porous spheres were transformed into NaO_2_, which decomposed into Na^+^ with O_2_ release. This study revealed the structural changes associated with the electrochemical processes at the electrode/electrolyte interfaces, which constitutes the basis for further visualization of the interfacial chemical species.

### Visualization of the Formation of Lithium Dendrites and SEI Films

The morphological and structural evolution induced by dendrite growth and the formation of SEI at the electrode–electrolyte interface are closely related to the cyclic stability of LIBs [92, 93]. The growth of lithium dendrites can lead to short circuits in batteries, resulting in serious safety issues and poor stability. The continuous growth of the SEI films after cycling results in significant overpotentials on the anode materials (Si, graphite, Li metal, etc.), thereby shortening battery lifespan [94]. Addressing these issues requires a comprehensive understanding of the growth mechanisms of both lithium dendrites and SEI films. But both are highly sensitive to water and oxygen in the air, ex-situ characterization is far from sufficient. Therefore, in-situ TEM technologies have been developed to capture these unstable intermediates in real time.

**Fig. 4:**
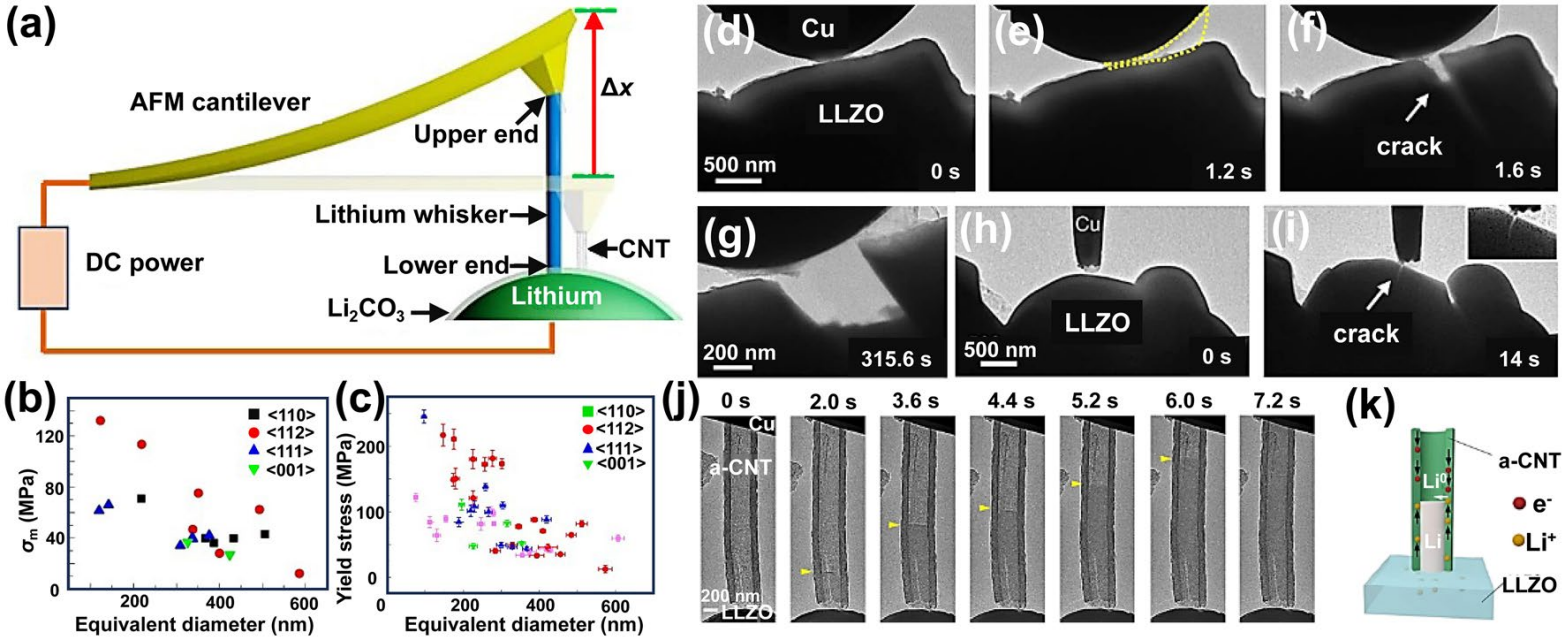
In-situ visualization of the formation of interfacial chemical species. **a** Scheme of an in-situ AFM–ETEM device for observing and measuring Li whisker growth. **b, c** Plots of the maximum stress (*σ*_*m*_, **b**) and yield stress (**c**) of Li whiskers in different growth directions as a function of the equivalent diameter. Reproduced with permission [80], Copyright 2020, Springer Nature. **d-g** In-situ TEM images of a LLZO particle ruptured by Li eruption at the interface. **h, i** Li bursts puncture and short-circuits one LLZO particle that is in close contact with adjacent particles. **j** In-situ TEM image of rapid Li plating in amorphous carbon nanotubes (a-CNT). The leading edge of lithium growth is marked with a yellow arrow. **k** Scheme of a-CNT wall serves as the host of Li. Reproduced with permission [96], Copyright 2022, Springer Nature

As the ultimate anode material for future LIBs, lithium metal faces uncontrollable Li dendrites growth, which limits the progress of Li metal-based LIBs [94, 95]. Zhang et al. investigated the growth process and stress characteristics of lithium whiskers through combined experiments using in-situ AFM and ETEM (Fig. 4a) [80]. It was found that under the applied electric potential, lithium dendrites grew between the tip and the Li metal, generating stress as high as 130 MPa (Fig. 4b). Additionally, the study measured the yield strength of lithium dendrites under mechanical loading, which reached 244 MPa (Fig. 4c). These results provide quantitative indicators for designing strategies to constrain the growth of lithium dendrites. Gao et al. revealed the growth and evolution of lithium dendrites at the interface of solid electrolyte Li_7_La_3_Zr_2_O_12_ (LLZO) through in-situ TEM [96]. Under the condition where the current collector (Cu probe) was fixed by external force, the rapid growth of lithium dendrites created a huge stress at the contact region, leading to the formation of cracks in the solid electrolyte (Fig. 4d-i). The dendrites then grew along these cracks, resulting in the transgranular fracture of the entire LLZO particle, ultimately causing the failure of solid electrolyte and short-circuit of battery. The use of a host with rapid lithium storage capability (as a buffer layer between the current collector and the solid electrolyte) facilitates uniform lithium deposition, prevents damage to the solid electrolyte caused by lithium deposition, enabling rapid charging of solid-state batteries. In-situ TEM observations reveal that the Li metal rapidly fills the cavities of amorphous carbon nanotube by Li^+^ diffusion along the carbon shells with a large current density (Fig. 4j, k) [96]. This implies that at room temperature, the Li transport via Li^+^ diffusion along the carbon-based host is more effective than the Li creep, thereby preventing damage to the solid electrolyte surface.

**Fig. 5:**
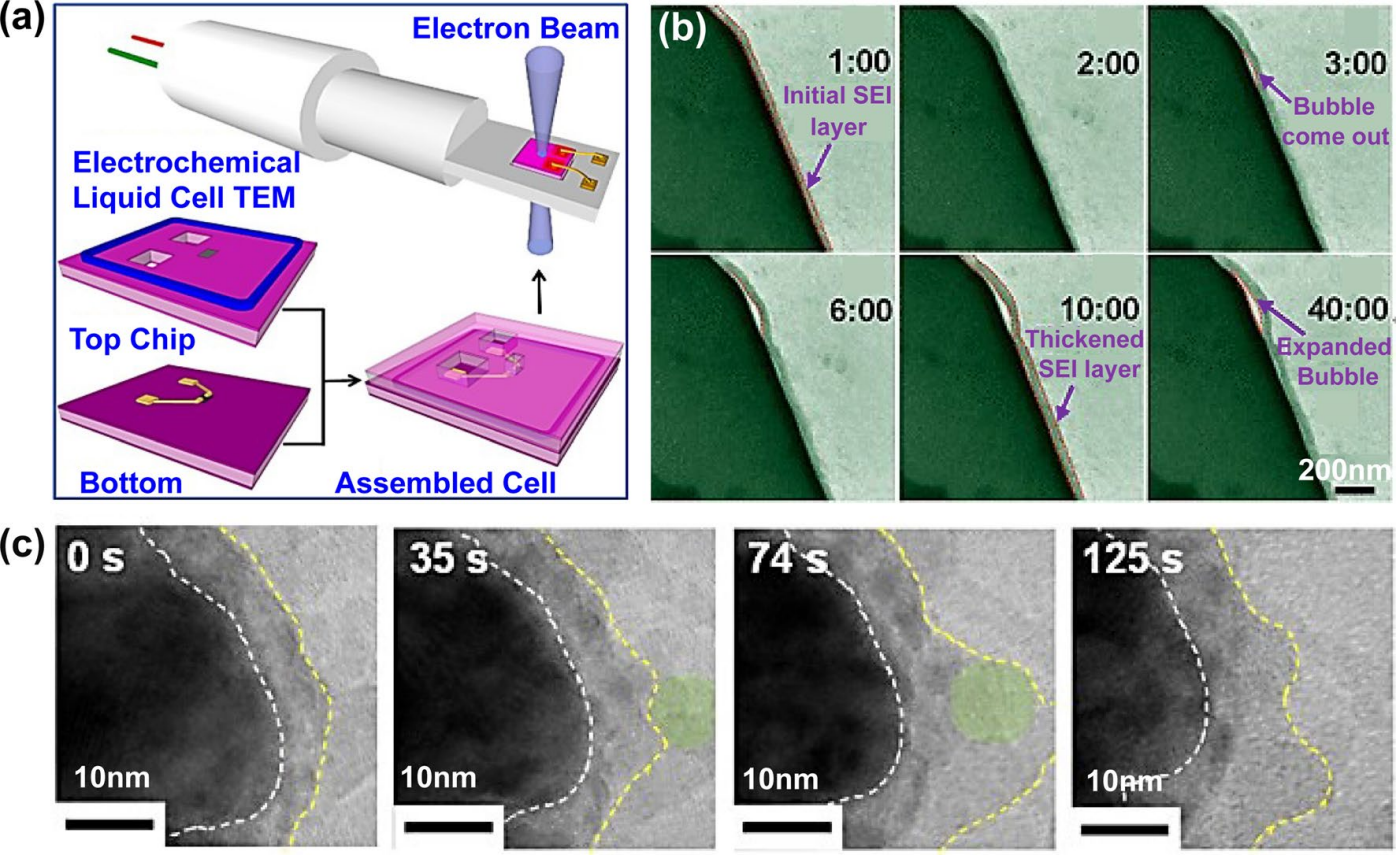
**a** Scheme of an electrochemical liquid cell. **b** In-situ TEM images of electrochemical reaction show the growth of a SEI film. Reproduced with permission [84], Copyright 2014, American Chemical Society. **c** Morphological observation of SnO_2_ surface analyzed by TEM using graphene liquid cell. Reproduced with permission [97], Copyright 2016, Elsevier

The formation of SEI films is essential for enhancing the performance of energy storage devices. To observe the solid electrolyte layer growing in real electrochemical reactions, Zeng et al. developed an electrochemical liquid cell for in-situ TEM observation (Fig. 5a) [84]. Benefiting from this advanced technology, the dynamic lithiation of Au electrodes in commercial LiPF_6_/EC/DEC electrolyte was captured, including Li metal dendritic growth, electrolyte decomposition, as well as SEI formation. During the initial lithiation stage, electrolyte decomposition generated bubbles on the electrode surface, indicating that the solid electrolyte interphase is beginning to form (Fig. 5b). When the SEI thickness reached about 200 nm, the SEI growth rate significantly slowed down and lithium dendrites began to appear on the SEI surface. In the subsequent lithiation process, lithium dendrites were transformed into dead lithium and adhered to the surface of the membrane due to dissolution. Benefiting from the development of graphene liquid cell, the detailed formation process of the SEI on SnO_2_ nanotubes was observed through in-situ TEM. This process involved the simultaneous decomposition, deposition, and stabilization of the electrolyte during lithiation (Fig. 5c) [97]. During the initial formation stage, the reduction products of the electrolyte deposited to form a thin interfacial layer under electron beam irradiation. At the deposition stage, the decomposed electrolyte aggregated and deposited into the SEI layer, resulting in uneven thickness of the SEI layer. Meanwhile, the decomposed electrolyte deposited on the SnO_2_ nanotubes at different times, concurrently with the stabilization process of the SEI layer.

**Fig. 6:**
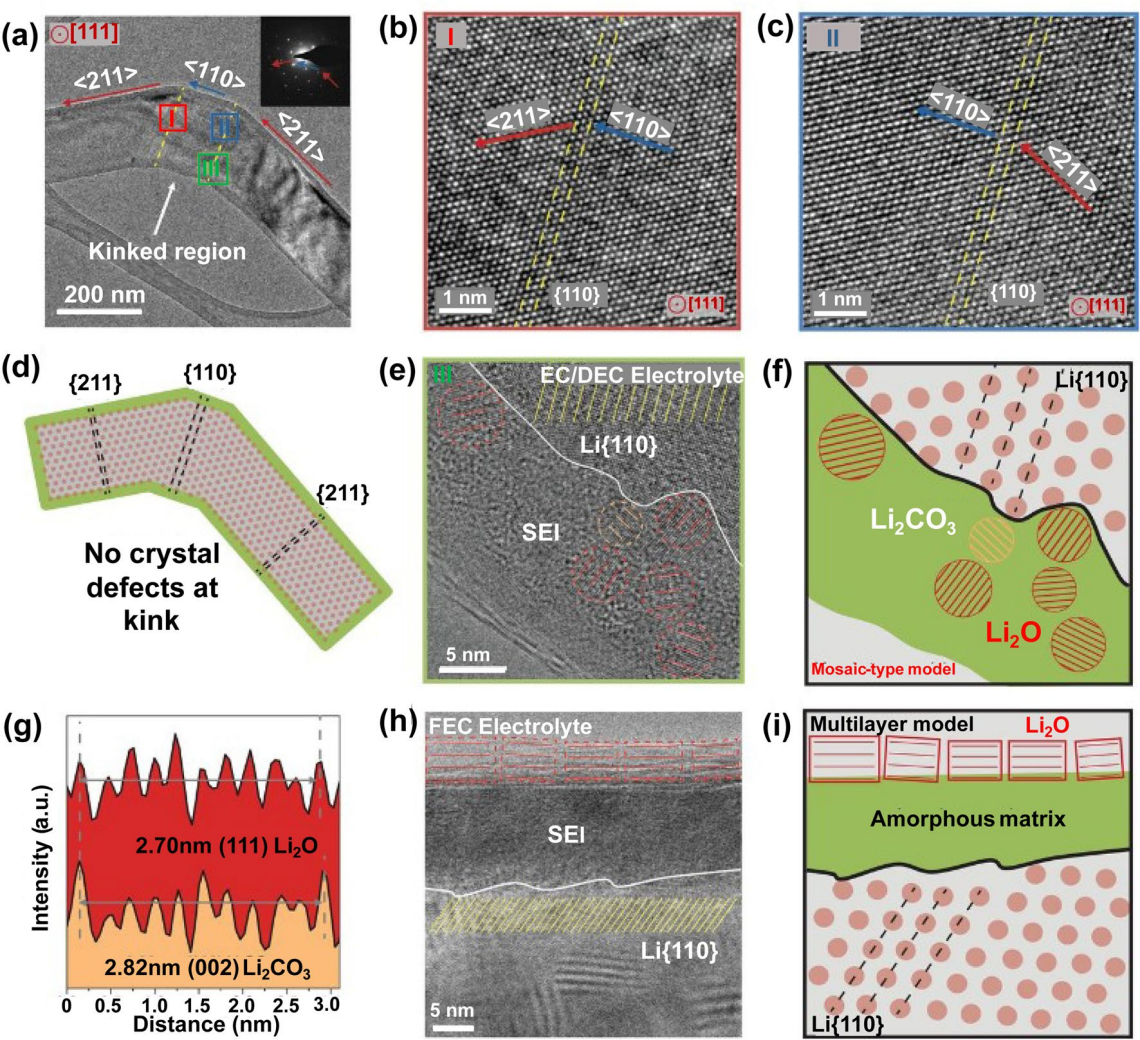
**a–c** TEM image (**a**) and magnified views (**b, c**, see color labeled) of lithium dendrite in different growth direction. Inset in (**a**): SAED pattern. **d** Schematic of the lithium dendrite. **e** HRTEM image shows the green region labeled in (**a**, III). The lattice spacings of small crystalline grains in the amorphous film match those of lithium carbonate (orange circles) and lithium oxide (red circles). **f** Schematic diagram of the embedded structure on lithium dendrites in electrolyte. **g** The integrated pixel intensity of lithium oxide (red) and lithium carbonate (orange) lattices. Peaks and valleys correspond to atomic planes and gaps, respectively. **h** HRTEM image of a different SEI in FEC electrolyte. **i** Scheme of the multilayer structure on lithium dendrites. Reproduced with permission [100], Copyright 2017, American Association for the Advancement of Science

One of the significant observations is the gradual stabilization of the SEI layer, forming an increasingly uniform structure at the interface. This stage primarily involves reducing excess interfacial energy to decrease the thickness of the SEI layer. This finding is clearly benefited from advanced in-situ TEM techniques, allowing visualization of the microscale growth processes of lithium dendrites and SEI films. While it provides a scientific basis for the development of strategies to address interfacial degradation behavior, an in-depth understanding of the interfacial mechanistic details in terms of electronic structures and chemical composition is needed, especially by utilization of some other related techniques with both structural and composition sensitivities. In fact, lithium dendrites and SEI layer are extremely unstable under the electron beam. Their formation can be observed using in-situ TEM in the low-resolution mode by reducing the beam radiation dose. To overcome the challenge, cryo-TEM technique was developed to investigate the formation and lattice structure of lithium dendrites and SEI [98–100]. For example, lithium dendrites in carbonate-based electrolytes were found to grow as single-crystal nanowires along the < 111 > (preferred), < 110 > , or < 211 > directions (Fig. 6a-d) [100]. In addition, the information on composition and structure of SEI layer in different electrolyte were obtained. In the widely used carbonate-based electrolyte, SEI layer exhibits a non-uniform distribution of organic and inorganic components, showing particles of lithium oxide and lithium carbonate dispersed within an amorphous matrix (Fig. 6e-g). In contrast, the SEI formed in a carbonate-based electrolyte containing a certain amount of fluoroethylene carbonate (FEC) is more ordered and resembles a multilayer (Fig. 6h-i). The inner layer is primarily amorphous, while the outer one consists of large grains of lithium oxide with distinct lattice fringes.

## Use of EELS for Characterization of Interfacial Mechanisms

### Capabilities of EELS Characterization

A thorough comprehension of the changes in the chemical composition of electrode materials as they undergo electrochemical reactions is essential to grasp the fundamental mechanisms of battery charging and discharging. To achieve this, various advanced characterization techniques have been extensively employed in the field of chemical compositional analysis. These techniques include, but are not limited to, EELS, soft and hard X-ray absorption spectroscopy (XAS), energy dispersive X-ray spectroscopy (EDX), and Raman spectroscopy [101, 102]. Each technique offers unique advantages for elucidating the composition changes during electrochemical reactions. Among them, EELS stands out for its unparalleled high spatial resolution at the atomic level [103, 104]. This superior resolution is significantly enhanced by the monochromators and spherical aberration correctors, allowing for much greater accuracy in analyzing the changes in chemical composition.

EELS is particularly valuable as it provides comprehensive information not only on the element types, valence states, and concentration distributions, but also details on the coordination environments within specific lattice regions [105]. To facilitate this level of analysis, EELS instruments are typically coupled with TEM or its scanning counterpart, scanning transmission electron microscopy (STEM). Such configurations enable the EELS to be merged with a wide array of multifunctional specimen holders, thereby empowering the technique with the capability to perform in-situ characterization under diverse external fields. This is a significant advantage as it allows for the analysis of chemical composition changes in real-time, without disrupting the electrochemical reaction. The advancement of in-situ EELS technology has proven to be a great boon to research on battery charging and discharging mechanisms, as it has significantly promoted our understanding of the complex variations in the chemical composition of electrodes in intricate electrochemical reactions.

### Characterization of the Electronic Structure

**Fig. 7:**
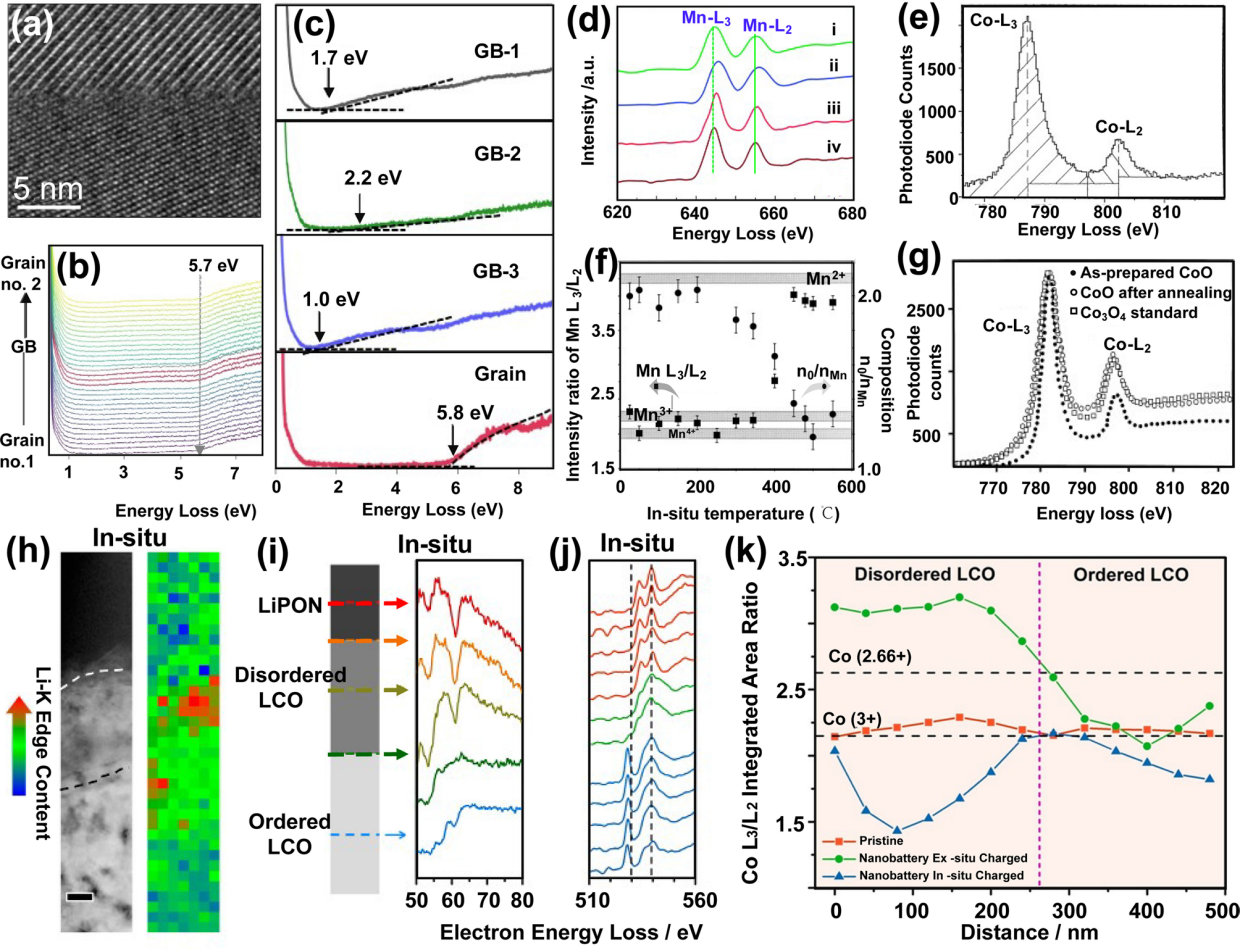
**a** HRTEM imaging and analysis of GB (grain boundary) of pristine LLZO. **b** Line-scan EELS of GB. **c** Bandgap measurements. Reproduced with permission [109], Copyright 2021, Springer Nature. **d** EELS patterns of specimens Li_1.2_Ni_0.2_Mn_0.6_O_2_ (i), Li_1.2_Ni_0.19_Mn_0.59_Co_0.02_O_2_ (ii), Li_1.2_Ni_0.18_Mn_0.58_Co_0.04_O_2_ (iii), and Li_1.2_Ni_0.15_Mn_0.55_Co_0.1_O_2_ (iv). Reproduced with permission [68], Copyright 2014, Royal Society of Chemistry. **e** An EELS spectra acquired from a Co oxide, showing the technique used to extract the intensities of white lines. **f** Plots of Mn L_3_/L_2_ and the chemical composition of n_O_/n_Mn_ based on EELS spectra. **g** A comparison of EELS spectra of Co–L_2,3_ ionization edges. Reproduced with permission [111], Copyright 2000, Elsevier. **h-j** STEM image and EELS characterization: (**h**) High-angle annular dark field (HAADF) image of the nanobattery stack along with Li K-edge concentration mapping. **i-j** Li K-edge (**i**) and O K-edge (**j**) spectra from the disordered/ordered LCO. **k** Ratio analysis. Reproduced with permission [81], Copyright 2016, American Chemical Society

EELS provides valuable information about the electronic structure of a sample by measuring the electron energy loss during the interaction of the electron beam with sample [105, 106]. By analyzing the energy loss spectrum, electronic structure such as the electronic energy level structure, bandgap width, and valence state distributions of the sample can be inferred. In an EELS spectrum, the valence region represents the energy loss caused by the valence electrons jumping to the conduction band, and its lowest value corresponds to the band gap [107–109]. For example, Liu et al. studied the transport properties of Li^+^ in grain boundaries within LLZO by conducting in-situ STEM and EELS (Fig. 7a-c) [109]. The grain boundaries show a reduced band gap in comparison with that for bulk LLZO, which induces the formation of lithium dendrites within the grain boundaries as the flow of electrons through the grain boundaries.

Detecting changes in the valence states of elements in electrode materials is commonly used for electrochemical kinetic studies. Huang et al. demonstrated via atomic-level EELS that the Co doping increases the valence states of Mn within Li_1.2_Ni_0.18_Mn_0.58_Co_0.04_O_2_, thereby inhibiting the Mn^3+^/Mn^4+^ induced Jahn–Teller effect [68]. As shown Fig. 7d, as the amount of Co doping increases, the edge of Mn–L shifts toward higher energy loss positions for higher oxidation, which agrees with the general chemical shift rule [68, 110]. For 3*d* transition metal elements, their valence states can be quantified by analyzing their L edges [111, 112]. The L_3_ and L_2_ edges follow transitions of 2*p*^3/2^ → 3*d*^3/2^3*d*^5/2^ and 2*p*^1/2^ → 3*d*^3/2^, respectively, while each intensity is associated with an unoccupied state in the *3d* bands. By calculating the L_3_/L_2_ integrated area ratio, the valence states can be revealed [111–113]. In an earlier report, Wang et al*.* applied EELS to quantitatively determine the valence states of Mn and Co oxides by calculating the L_3_/L_2_ integrated area ratio, which provided a way to measure the valence states of Co or Mn (Fig. 7e-g) [111]. However, the redox couple of Co^3+^/Co^4+^ is difficult to detect by ex-situ measurements due to the unstable Co^4+^ in LiCoO_2_ (LCO) cathode. Coupling EELS with in-situ TEM technology (Fig. 7h, i), the valence changes of Co in the LiCoO_2_ cathode were tracked in real-time. The operando EELS results indicated that the oxide of Co changed from initial Co^3+^ to Co^4+^ (Fig. 7j, k) [81].

**Fig. 8:**
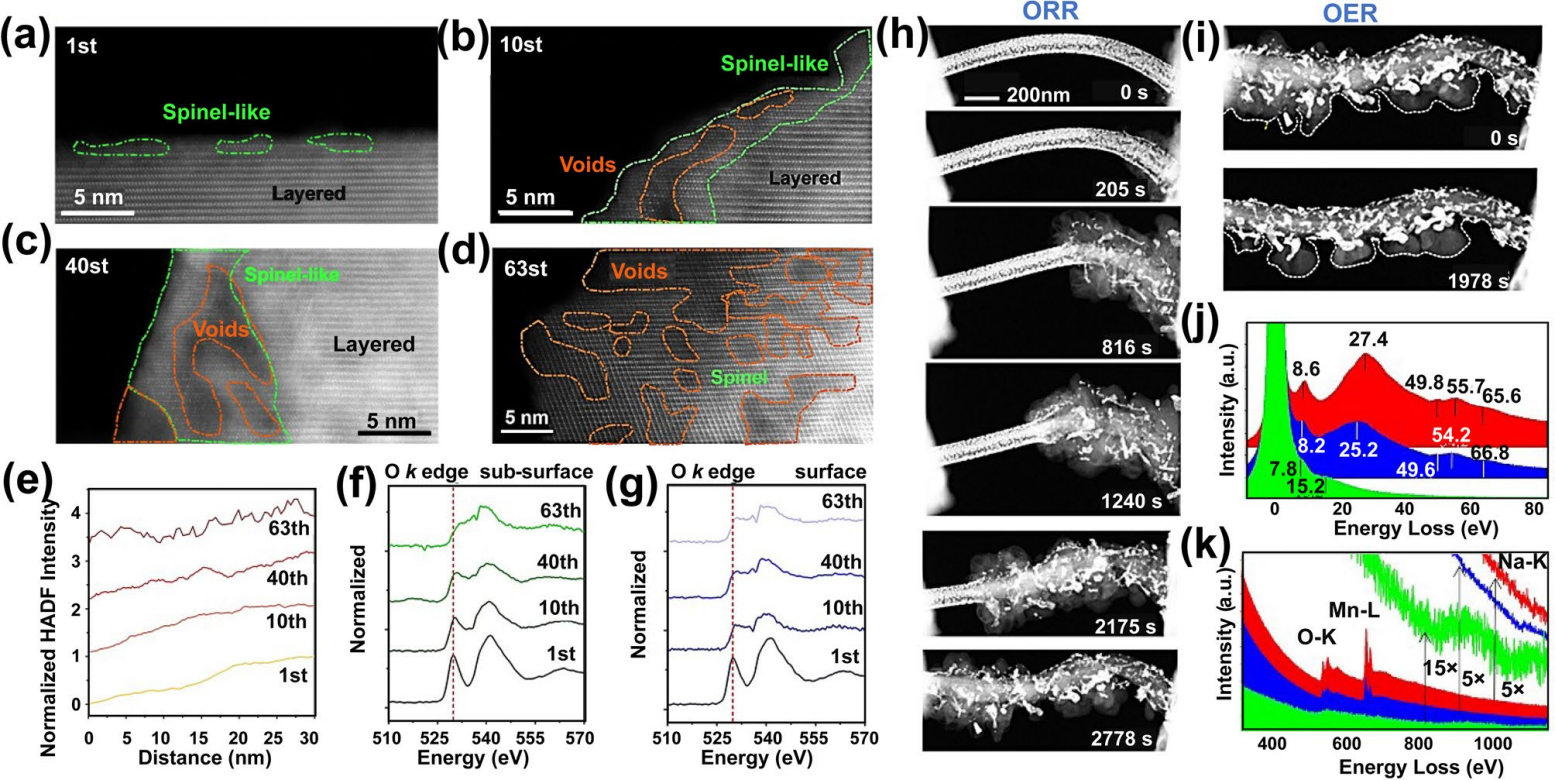
STEM and EELS in in-situ imaging of interfacial structure evolution. **a-d** HAADF images show the evolution of nanovoids and the formation of spinel structures during extended cycling. **e** STEM-HAADF intensity distribution of particles with different cycling times. **f, g** EELS results of O-K edge obtained from the sub-surface and surface. Reproduced with permission [116], Copyright 2023, Elsevier. **h** In-situ imaging the structure evolution of the NaO_2_ discharge product during ORR. **i** Charge process of the Au/MnO_2_ NW during OER. **j** Low-loss spectra. The major plasmon peak shifts from the pristine Au/MnO_2_ NW (red) to the NaO_2_ layered on Au/MnO_2_ nanowires (blue), to NaO_2_ (green). **k** Core-loss spectra. Au/MnO_2_ (red) shows the presence of Mn and O, while the discharge products (blue) reveal the presence of Na in addition to Mn and O, as well as weak O–K and Na–K edges. Reproduced with permission [117], Copyright 2019, Elsevier

Benefit from high spatial resolution, EELS has been used to uncover the formation of oxygen vacancies and oxygen redox reactions in cathode materials [114]. As an example, Yan and coworkers investigated the chemical composition of the degradation layer on the surface of cycled Li_1.2_Mn_0.6_Ni_0.2_O_2_ using EELS. The EELS results revealed that the formation of oxygen vacancies led to an irreversible phase-transition from hexagonal *R-3C* to cubic *Fd-3m* in Li_1.2_Mn_0.6_Ni_0.2_O_2_ cathode [115]. Zhang et al. used ex-situ TEM and EELS to investigate the structural evolution of Li_*x*_CoO_2_ (Fig. 8a-d) [116]. As shown in Fig. 8e-g, the O pre-edge at 530 eV depresses is accompanied by a transition of hexagonal *R-3c* → cubic *Fd-3m* → cubic *Fm-3m*, suggesting that the oxygen loss induces structural degradation of Li_*x*_CoO_2_. In combination with ETEM, the oxygen reduction mechanism in Na–O_2_ batteries was investigated by in-situ EELS [117]. According to the in-situ results, the NaO_2_ intermediates on the Au-coated MnO nanowires were decomposed into Na_2_O_2_ and O_2_ during the oxygen reduction process, which resulted a decrease of O pre-edge (Fig. 6h-k).

It is evident that the combination of in-situ TEM and EELS techniques enables real-time monitoring of the evolution of both nanostructural and chemical composition of electrode materials during electrochemical reactions. This provides crucial scientific evidence for in-depth understanding of the electrochemical reaction mechanisms, which calls for further determination of the dynamic processes at the interfaces.

### Characterization of Interfacial Dynamic Ion Transportation

For LIBs, the Li^+^ ions inside electrodes are difficult to directly observe by X-ray-based technologies, such as XPS, XAS, and EDS because of the low energy region of Li (~ 60 eV). EELS is highly sensitive to light elements (H, He, Li, and B, etc.) as the outer electrons of light elements with low binding energy are more easily to be excited by low-energy incident electrons, resulting in more pronounced energy loss signals [118]. Therefore, operando STEM/TEM combined with EELS has been widely used to investigate the dynamical behaviors of Li^+^ ion transport within operating LIBs batteries [119].

**Fig. 9:**
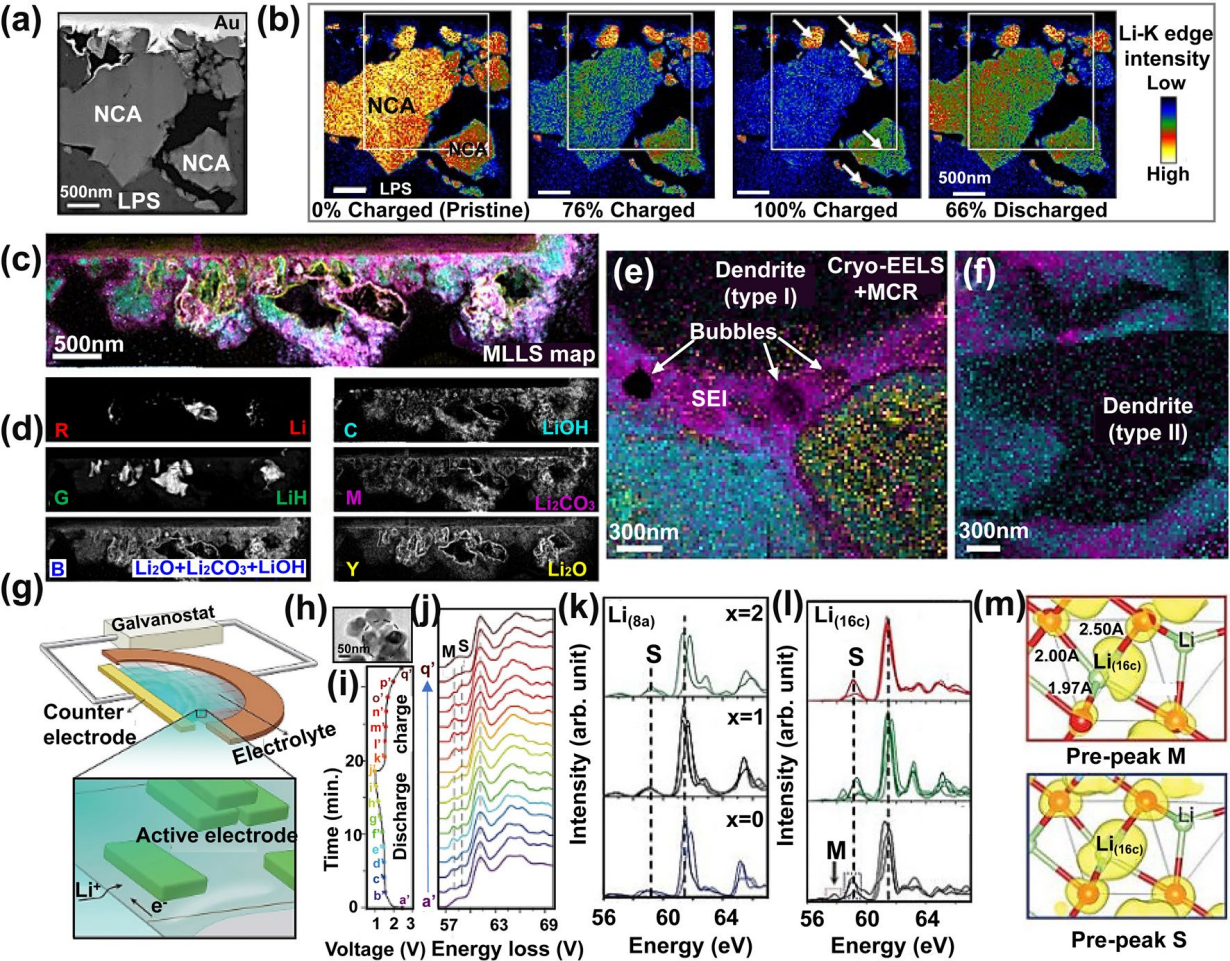
ADF-STEM and EELS in-situ imaging of SEI. **a** Annular dark-field STEM (ADF-STEM) image of electrochemically active region of thin-film solid-state cell. **b** Li-concentration maps of Li_x_Ni_0.8_Co_0.15_Al_0.05_O_2_ polycrystalline particles at different charge and discharge states. Reproduced with permission [120], Copyright 2020 American Chemical Society. **c** EELS map decomposed by multiple linear least-square (MLLS). **d** Corresponding grayscale EELS-MLLS images showing the individual components of (**c**). Reproduced with permission [121], Copyright 2023, American Association for the Advancement of Science. **e, f** Structure and elemental composition analyzed by cryo-electron TEM. Reproduced with permission [122], Copyright 2018, Springer Nature. **g** An electrochemical cell based on ionic liquid electrolytes. **h-j** TEM image (**h**), corresponding EELS spectra (**i**), and intensity map of Li-EELS spectra (**j**) for Li_4_Ti_5_O_12_ nanoparticles. The vertical dashed black lines indicate the energy positions of the main peaks at ~61.5 eV and pre-peaks M (related to metastable configurations of the intermediate compositions) and S (related to stable configurations in Li_4_Ti_5_O_12_ and Li_7_Ti_5_O_12_). **k, l** Calculated Li-EELS spectra of Li_4+*x*_Ti_5_O_12_ at Li 8a (**k**) and 16c (**l**) sites. **m** Isosurface plot of partial charge density associated with face-sharing Li(16c) in Li_5_Ti_5_O_12_ in two energy ranges. Reproduced with permission [124], Copyright 2020, American Association for the Advancement of Science

By integrating the intensity of the first peak in the Li–K edge spectra, Nomura et al*.* mapped the Li^+^ ion transport in electrochemical solid-state LiCoO_2_||LiPON||Li battery (Fig. 9a-b) [120]. The dynamic images of Li concentration and Co L_3_/L_2_ in LiCoO_2_ single-crystal particles at different charge states showed that the grain boundaries between nanocrystals have a significant impact on the lithium-ion transport, that is, lithium ions were preferentially transported between particles with consistent lattice orientation. By optimizing the orientation relationship of nanocrystals, reducing lattice mismatch, and improving the tightness of nanocrystal interfaces, the high-rate capability of solid-state LIBs can be effectively improved. It is important to note that the SEI is a key component in LIBs, while revealing the evolution of its chemical composition under high electronic radiation remains a challenge. High collection efficiency, EELS is considered capable of operating at low doses to minimize beam damage [105]. In the study by Lodico et al. using STEM and EELS technology to track the charging and discharging process of electrochemical fluid LIBs, the growth and changes of SEI were observed (Fig. 9c, d) [121]. The EELS mapping showed that the SEI layer was mainly composed of various lithium compounds (Fig. 9d), including lithium, lithium hydride, lithium oxide, lithium carbonate, lithium hydroxide, etc. By using cryogenic transmission electron microscopy (cryo STEM) and EELS techniques, Zachman el al. analyzed the structure and chemical composition of the solid–liquid interface and dendritic structure in lithium metal batteries (Fig. 9e, f) [122]. The results indicated that there were extended solid–liquid interface layers and LiH dendritic structures on the anode of lithium metal batteries, while carbonate structures existed in some lithium fluoride electrolytes. In addition, researchers have found that the introduction of fluorinated electrolytes can effectively suppress the formation of LiH dendritic structures and improve the performance of batteries.

The coordination environment of Li in the lattice can be reflected by Li–K edge. The pre-edge of the latter is highly sensitive to the local coordination of lithium, reflecting the Li occupancy and coordination of lithium [123]. Based on it, Zhang et al. built an ionic liquid electrolyte LIB cell for operation inside a TEM, enabling operando EELS to probe the Li occupancy and transport in Li_4_Ti_5_O_12_ during electrochemical cycling (Fig. 9g-j) [124]. The Li K-edge of Li_4_Ti_5_O_12_ composed a broad peak in the post-edge region (~ 58.9 eV), which mainly came from the inelastic scattering of Li at 8*a* sites. In the discharge process, a new pre-peak appeared in the post-edge region (~ 58.0 eV), implying distortion of the Li–O bond. Besides, DFT calculations showed that the Li–O bonds in the LiO_6_ octahedron at 16c site is elongated when Li_4_Ti_5_O_12_ is lithiated to Li_5_TiO_12_. This process weakens the Li–O bond and lowers the  energy state of the anti-bond Li–O, resulting in pre-peak splitting. The findings demonstrate that the Li^+^ ions in the Li_4_Ti_5_O_12_ migrate from their initial tetrahedral 8*a* sites to the octahedral 16*c* sites, which contributes to the fast-charging behavior (Fig. 9k-m).

The understanding of the dynamic ion transportation clearly benefited from coupling EELS’ capability, especially in detecting light elements and Li-ions inside operating LIBs. This provides an important insight into the general mechanistic processes in ion transport, but the detection of the mechanistic details would benefit from improved image resolution in the in-situ studies.

## Electron Holography for Detection of Dynamic Ion Transport

Recently, many studies have shown that electron holography, a powerful imaging technique used in electron microscopy, enables the visualization of phase information in electron waves, providing insights into electromagnetic fields, potential distributions, and material properties at the nanoscale [125–129]. The evolution of electric fields and potentials in complex electrochemical reactions is usually induced by ionic migration and charge exchange [130]. Visualization of electric potential distributions would help analyze electrochemical reactions during cycling and inform the development of safer, cheaper, and more efficient batteries.

**Fig. 10:**
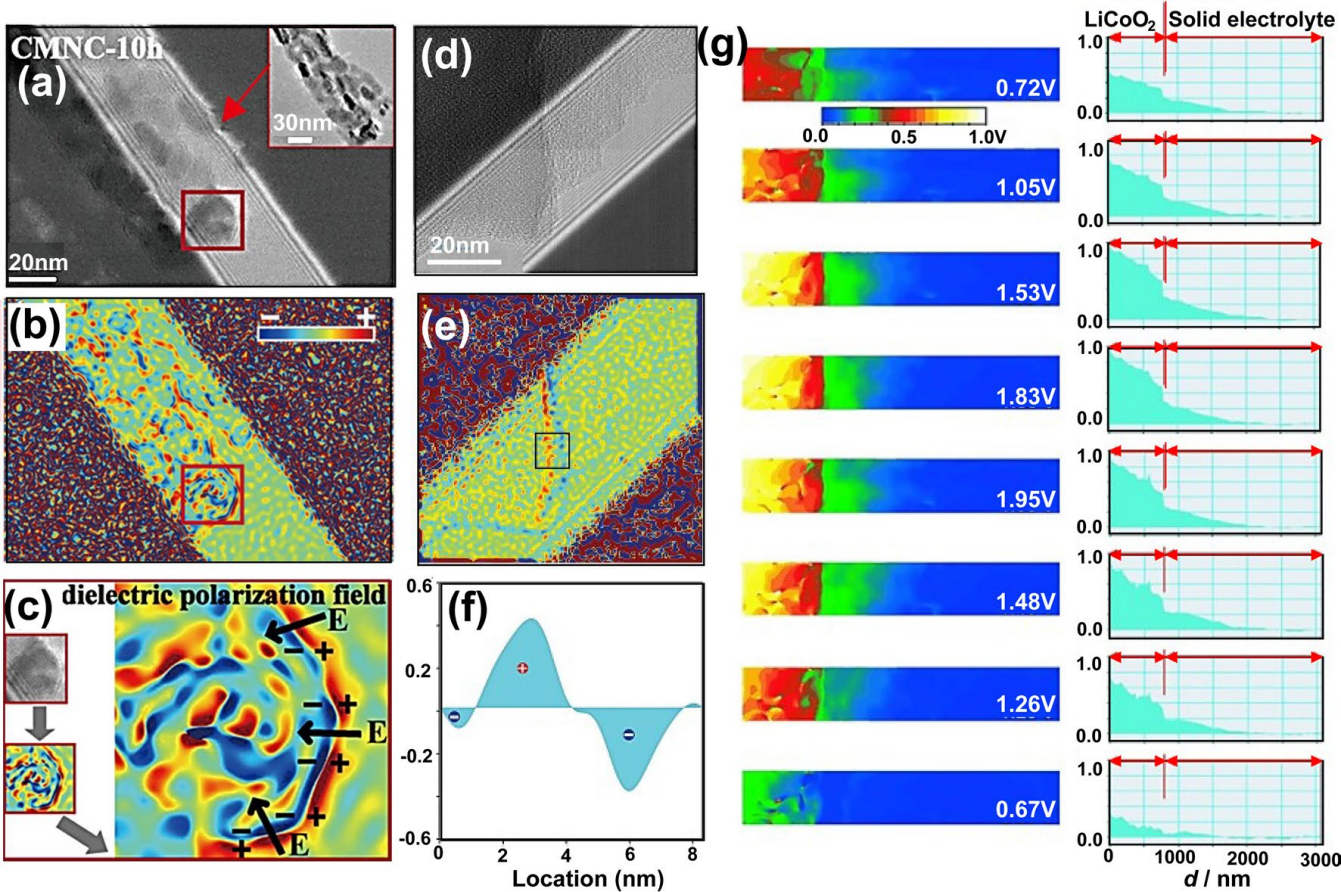
Electron holography imaging of electrode/electrolyte interfaces. **a-c** The electron holography image (**a**), charge density map (**b**), and dielectric polarization field (**c**) of Mn/Ni co-doped CoO/C hollow microspheres (CMNC-10h, the hydrothermal time was set at 10 h). Reproduced with permission [69], Copyright 2019, American Chemical Society. **d-f** The electron holography (**d**), the corresponding charge density map (**e**), and the averaged charge density profiles (**f,** from the black rectangular) of Ce_1/3_NbO_3_ surface. Reproduced with permission [67], Copyright 2022, Wiley. **g** 2D images (left) show the distribution of electric potential around the LiCoO_2_/electrolyte interface during the cycling process; right: line profiles. Reproduced with permission [134], Copyright 2010, Wiley

For electrode materials based on insertion chemistry, crystal defects significantly impact their intrinsic ion transport and storage properties. Although crystal defects can be observed or detected by HAADF imaging or other measurement methods such as XRD and AFM, it remains challenging to explain the mechanism by which defects affect ion transport and storage [131]. In recent decades, electronic holography has been conducted for revealing the microstructure of electrode materials, including crystal defects, phase separation, and particle interface [132]. For instance, by using electronic holography, Li et al. successfully visualized the charge distribution and space charge layer on the surface of carbon-coated CoO hollow microspheres (Fig. 10a-c) [69]. Oxygen vacancies on the surface of nanoparticles caused the redistribution of surface charge (Fig. 10c), resulting in the formation of a space charge layer that accelerated the Li-ion mobility. Similarly, Liang et al. unveiled the surface-dependent sodium storage behavior in the cation-defected perovskite oxide Ce_0.333_NbO_3_ using electronic holography (Fig. 10d-f) [67]. The presence of cationic defect on the surface of CNO particles caused an accumulation of negative charges, which enhanced the adsorption of Na-ions on surface, thereby providing additional Na storage capacity.

It is widely recognized that the cathode/electrolyte interface contributes to high Li^+^-ion transmission impedance, which results in poor rate performance and capacity degradation of all-solid-state LIBs [133]. To visualize such interfacial resistance, in-situ electronic holography has been used for recording the evolution of electric fields and potentials at the cathode/electrolyte interface during electrochemical reactions. For instance, the potential distribution induced by lithium-ion diffusion at the interface of electrode–electrolyte in all-solid-state LCO/Li_1+*x*+*y*_AlyTi_2−*y*_Si_*x*_P_3−*x*_O_12_/Pt battery was directly observed through electronic holography (Fig. 10g) [134]. The electronic holography results demonstrated that during the charging process, the potential at the positive electrode gradually increased, while the potential in regions distant from the electrolyte/positive electrode interface remained nearly unchanged. This finding indicates that the resistance is predominantly localized near the electrolyte/electrode interface, especially within a region approximately one to two nanometers away from the electrolyte/electrode interface.

**Fig. 11:**
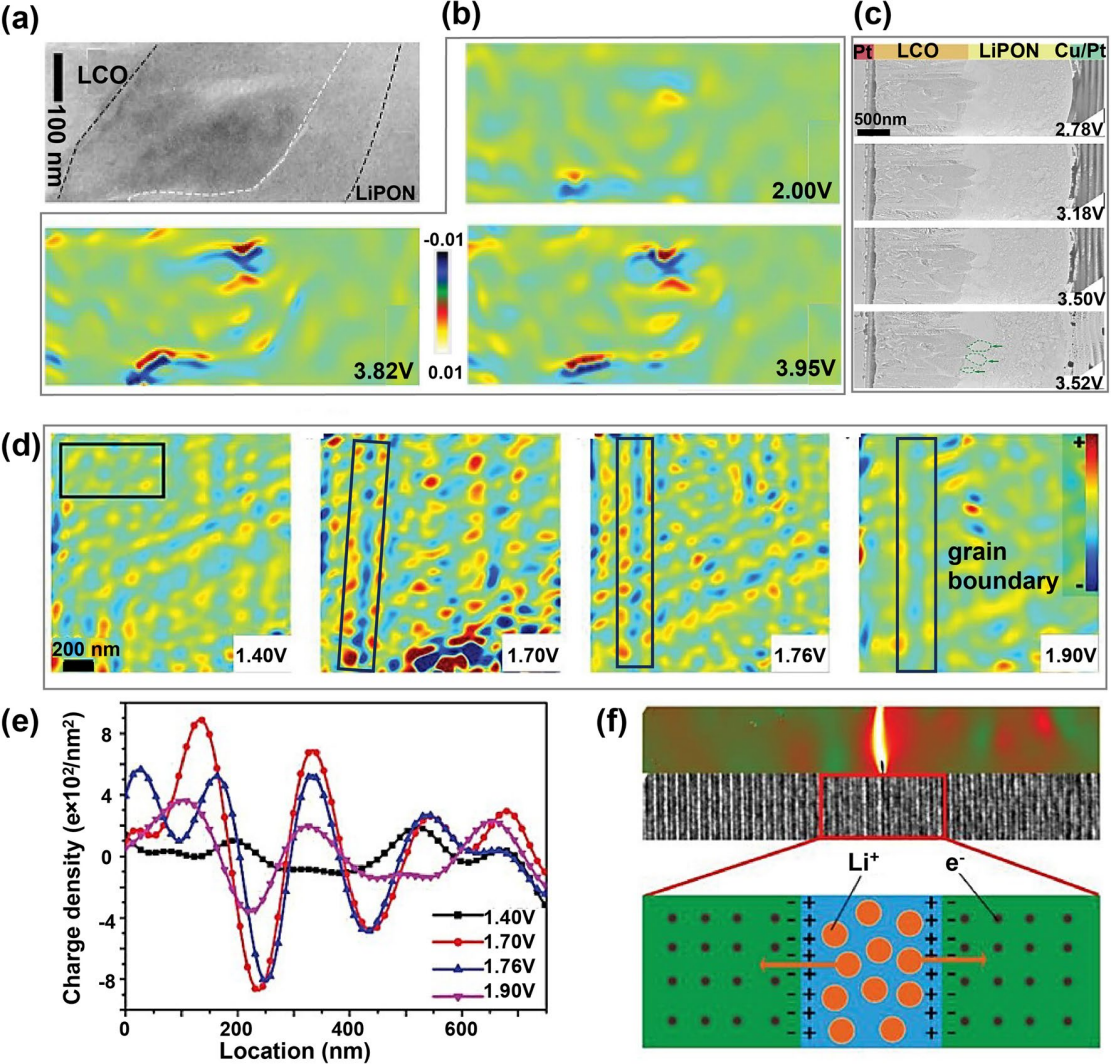
In-situ TEM imaging of ion transport at the interface. **a** TEM image shows the LiPON, LiCoO_2_, and interfacial layer. **b** Charge density distribution maps of (**a**) during cycling. **c** In-situ TEM images of the solid-state battery collected during charging process. Reproduced with permission [71], Copyright 2021, Wiley. **d** Charge distribution around grain boundary of Cu_2_Nb_34_O_87_ anode. **e** Averaged charge density curves from the black rectangular area in (**d**). **f** A diagram illustration of Li^+^ and electron distributions near the lattice strain region. Reproduced with permission [72], Copyright 2019, Wiley

Additionally, Yang et al. tracked the degradation behavior at the LiCoO_2_/LiPON interface in an all-solid-state LiCoO_2_/LiPON/Pt battery via in-situ electron holography coupled with in-situ TEM observation (Fig. 11a) [71]. During charging, the LiCoO_2_ near the LiCoO_2_/LiPON interface was corroded by LiPON, forming a degradation layer of 100 ~ 300 nm composed of a nanocrystalline layer and a transition layer. Electron holography results showed Li-ion accumulation at the interface boundary between the nanocrystalline layer and the transition layer (Fig. 11b). Moreover, in-situ TEM revealed that the nanocrystalline layer contained many voids, which gradually increased with prolonged charging time, leading to an insufficient Li-ion diffusion coefficient. The formation of voids within the nanocrystalline layer compromised structural stability and further elevated the Li-ion transfer impedance (Fig. 11c). Likewise, using in-situ electron holography, Yang et al*.* examined the migration of Li-ions along grain boundaries of Cu_2_Nb_34_O_87_ anode in real time (Fig. 11d-f) [72]. During lithiation, positively charged lithium ions preferentially propagated along grain boundaries, leading to an accumulation of positive charges at grain boundaries (Fig. 11d). Notably, once the lithium ions reached a critical concentration, they diffused into the surrounding lattice, visually elucidating the role of grain boundaries in enhancing lithium ions transport kinetics.

**Fig. 12:**
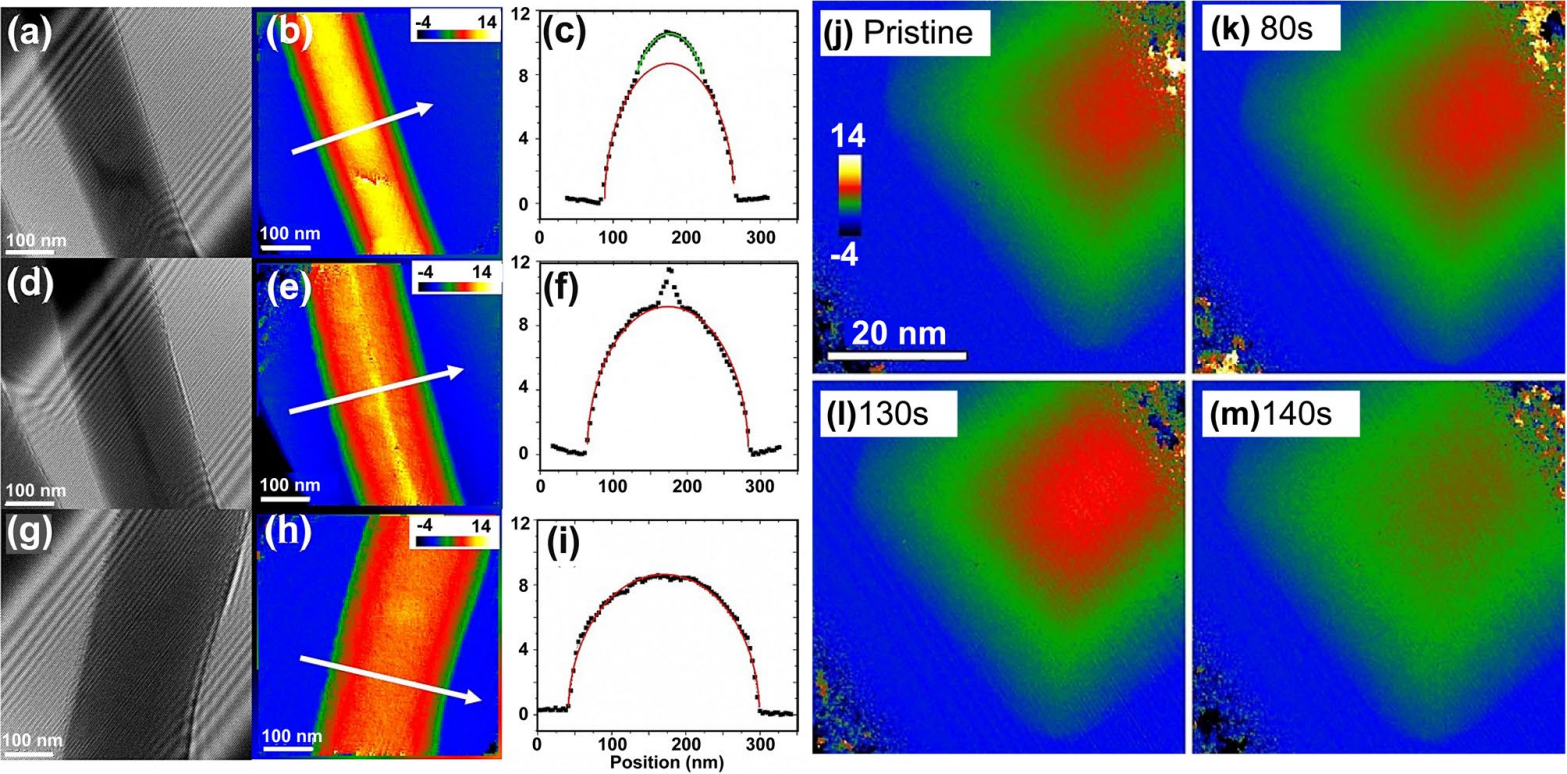
Electron holography in in-situ study of interfacial phase transition. **a-i** Electron Holograms (**a, d, g**), reconstructed phase images (**b, e, h**), and phase profiles (**c, f, i**, as illustrated by middle white arrows) of Ge/Li_*x*_Ge core/shell nanowire observed during lithiation. Reproduced with permission [135], Copyright 2016, American Chemical Society. **j-m** Electron holography reveals the phase transition of lithium ions in Li_4_Ti_5_O_12_ particles: phase maps of (**j**) pristine, (**k**) 80 s, (**l**) 130 s, and (**m**) 140 s captured for lithiated Li_4_Ti_5_O_12_ particle after reconstruction. Reproduced with permission [136], Copyright 2018, Elsevier

In one example employing in-situ TEM and electron holography, Gan et al. directly mapped the charge distribution during the lithiation of Ge nanowires [135]. They noted that during lithiation, the average internal potential within the Ge core remained lower than its theoretical value, attributed to the accumulation of trapped charges at the Ge core surface (Fig. 12a-i). These findings provide a direct pathway for observing the dynamic variations in charge distribution and establish novel pathways for studying electrode kinetics during battery charging and discharging processes. In another report using in-situ electron holography coupled with EELS, Wen et al. determined the phase transition of lithium ions in Li_4_Ti_5_O_12_ particles, revealing unique phase transition characteristics and charge storage mechanisms in Li_4_Ti_5_O_12_ materials (Fig. 12j-m) [136]. During lithiation, the Li_4_Ti_5_O_12_ phase gradually transformed into the Li_7_Ti_5_O_12_ phase, and a Li_4_Ti_5_O_12_/Li_7_Ti_5_O_12_ interface was formed inside the particle, which captured nearby electrons to generate a space charge layer. The resultant conductive Li_7_Ti_5_O_12_ phase on the surface of particles facilitated ultrafast discharge capabilities. In addition, the built-in space charge layer at Li_4_Ti_5_O_12_/Li_7_Ti_5_O_12_ interfacial reduced the overpotential, thereby enhancing the lithium-ion conduction across the interface. Both the distinctive features of phase transformation in the two-phase Li_4_Ti_5_O_12_ system contribute to the natural ultrafast discharge capability.

One of the most unique aspects of electronic holographic imaging technology lies in its ability to visualize field distribution within electrode materials. The further integration of electron holography with in-situ TEM technology enables real-time characterization of ion transport and phase transformation during electrochemical reactions. This characterization can be further enhanced by analyzing the geometric phase features to gain profound insights into the nanostructure strain properties.

## Imaging Strain Fields Inside Electrode Materials by GPA Technique

### Strain Mapping of the Electrode Interfaces

Understanding the stress evolution in electrode materials during charging-discharging is of great significance to improve their performance. Rechargeable secondary batteries (Li/Na/K/Zn ions, etc.) based on insertion chemistry frequently suffer from anisotropic lattice strains and stresses generated during the insertion and extraction of Li/Na/K ions, which results in crystal structure fatigue including crack formation, oxygen loss, and irreversible phase transitions [137]. For LIBs, during the electrochemical insertion and extraction process, the strain mainly originates from the large lattice-parameter variations induced by the electrostatic interaction of Li–O–Li and O–O, as well as multiple phase transitions caused by the changes of Li^+^ concentration [138]. On the downside, mismatched lattice parameters between the dominated and coexisting phases, or substabtial unit-cell volume variations, would creat fractured phase interfaces that severely cause capacity degradation [139, 140].

As an advanced method to obtain the strain field, geometric phase analysis (GPA) has been widely used to detect microscopic strains in crystal lattices of materials and analyze the changes in lattice as well as stress distributions [141–147]. The fundamental principle of GPA involves performing a Fourier transform on high-resolution images, selecting two nonlinear vectors, and subsequently conducting inverse Fourier transform to obtain high-precision displacement and strain fields at the microscopic measurement scale [148]. Recently, GPA is widely applied to inverstigate the strain distribution of electrode materials under various operational states.

**Fig. 13:**
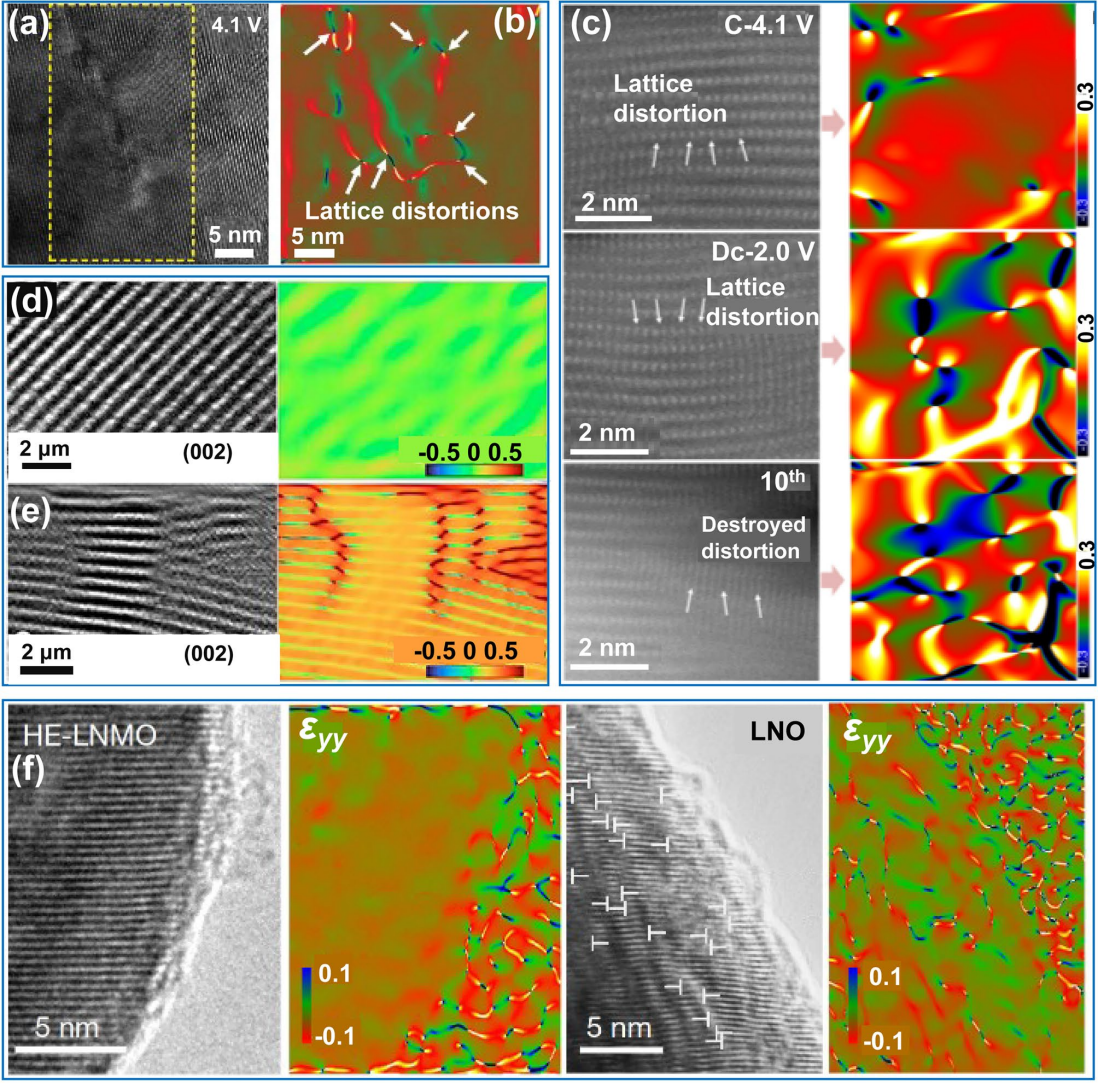
In-situ TEM imaging of strain evolutions. **a** TEM image of LiNi_0.83_Mn_0.06_Co_0.11_O_2_ charged to 4.1 V. **b** Strain state observed by GPA. Reproduced with permission [150] Copyright 2024, American Association for the Advancement of Science. **c** Lattice strain evolutions. Atomic-resolution HAADF-STEM images along the [010] zone axis and the corresponding ε_xx_ strain map obtained by GPA patterns for the Na[Ni_2/3_Ru_1/3_]O_2_. Reproduced with permission [153], Copyright 2024, Wiley. **d, e** The lattice distortion and strain images by GPA of P2–Na_0.8_Cu_0.22_Li_0.08_Mn_0.67_O_2_ (**d**) and P2–Na_0.8_Zn_0.22_Li_0.08_Mn_0.67_O_2_ (**e**). Reproduced with permission [154], Copyright 2023, American Chemical Society. **f** Strain state of the in-situ delithiated LiNi_0.8_Mn_0.13_Ti_0.02_Mg_0.02_Nb_0.01_Mo_0.02_O_2_ (HE-LNMO) and LiNiO_2_ (LNO) obtained by GPA. Reproduced with permission [155], Copyright 2022, Springer Nature

Currently, layered transition metal oxides are extensively employed as cathode materials for LIBs, NIBs, and KIBs, and they undergo multiple phase transitions during the delithiation process. Lattice displacements synergistically yield the build-up of phase transitions-driven lattice strains, which has been demonstrated as the primary cause of structural fracture [149, 150]. Huang et al. utilized GPA to reveal the severe lattice distortions formed in the lattice of Ni-rich layered oxide (LiNi_0.83_Mn_0.06_Co_0.11_O_2_). These lattice distortions intensified with the increasing delithiation depth, leading to the loss of oxygen and subsequent irreversible phase transition, where the initial layered structure transformed into a spinel structure (Fig. 13a, b) [150].

Compared with lithium ions, sodium/potassium ions with larger ionic radii cause more severe structural distortion and strain during their insertion and extraction from the host of the layered cathode material [151–153]. As shown in Fig. 13c, the insertion and extraction of sodium and potassium ions significantly alter the c-spacing of layered cathode materials, leading to rapid degradation of the lattice structure. Such lattice strain evolution can be effectively modulated through metal ion doping. For example, Cu doping reduced lattice distortion and strain by forming covalent Cu–(O–O) within the P2–Na_0.8_Cu_0.22_Li_0.08_Mn_0.67_O_2_ lattice (Fig. 13d, e) [154]. In addition, co-doping with Ti, Mg, Nb, and Mo in high-nickel layered materials significantly suppressed the cation mixing, phase transition, and oxygen loss, thereby reducing lattice strain and substantially enhancing cyclability (Fig. 13f) [155].

As stated above, GPA has become an important means to study the structural strain of electrode materials. Ex-situ GPA enables the analysis of structural distortion and strain distribution in electrode materials under specific conditions. However, some results fail to adequately explain the impacts of insertion and extraction behaviors of Li/Na/K ions on structural distortion and strain evolution, necessitating in-situ probing of dynamic lattice strain.

### Analysis of Dynamic Lattice Strain

As discussed in prominent examples, electrode materials exhibit intermediate states during electrochemical cycling that are  susceptible to structural transformations induced by exposure to oxygen and moisture in the air. Conventional ex-situ characterizations prove insufficient for inverstigating these  metastable intermediates in electrode materials. The development of in-situ TEM/STEM technologies has enabled  observation of structural evolution in the electrode materials in real-time at the atomic level. Importantly, in-situ TEM/STEM combined with GPA has been widely used to analyze the influence of lattice strains in intermediate states of electrode materials on structural stability [156].

**Fig. 14:**
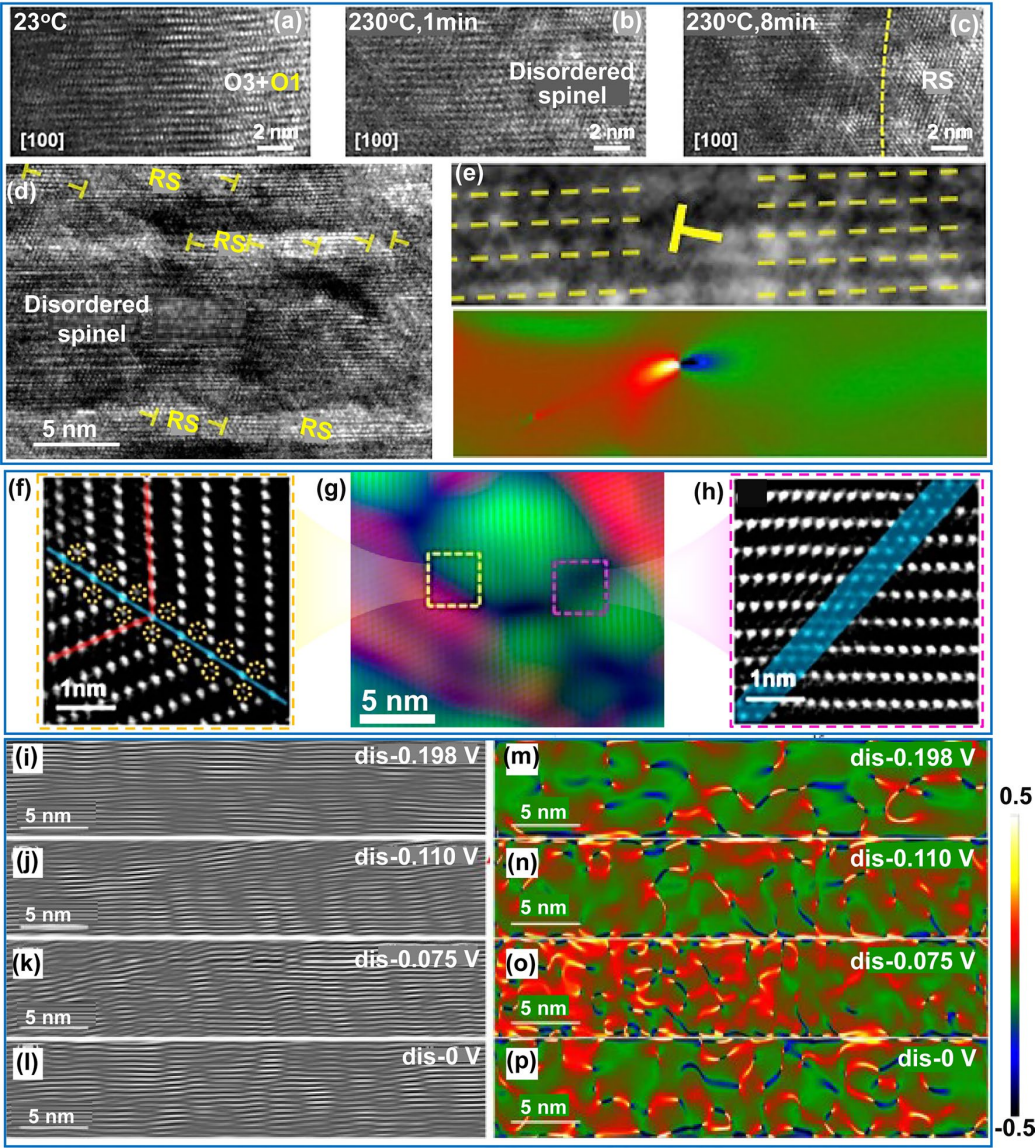
In-situ characterization of the dynamic lattice strains. **a-c** HRTEM images of the lattice evolution of delithiated Li_1−*x*_NiO_2_ during in-situ heating. **d** HRTEM image shows the dislocation with Burges vectors of 1/2 [110] configurated around crack tips. **e** The stress state of crack tip determined by GPA. Reproduced with permission [158], Copyright 2021, Elsevier. **f–h** HAADF image of the delithiated LiCoO_2_ cathode colored through the GPA method. The enlarged images show the yellow (**f**) and red (**h**) boxes in (**g**). Reproduced with permission [85], Copyright 2017, American Chemical Society. **i-p** Inverse FFT (fast Fourier transform) patterns (**i-l**) and strain maps (**m-p**) of graphite with different lithiation states at various potentials (dis: discharge). Colors: red → yellow → white: gradual increase in tension strain; green → blue → black: gradual increase in compressive strain. Reproduced with permission [159], Copyright 2023, Wiley

In practical applications, the thermal stability of electrode materials is critical for cycling and safety performance of batteries. Nickel-rich layered electrode materials at high charge states have been reported to exhibit poor thermal stability, often leading to structural and mechanical failures, such as phase transition, cation mixing, oxygen loss, and cracking [157, 158]. An example using GPA indicates that crack propagation is closely linked to the stress state at crack tips. The phase transformation induced by high temperatures and cation disordering leads to the formation of dislocations (Fig. 14a-c) [158]. Notably, such dislocations introduce a compressive strain field at crack tips (Fig. 14d). This stress field suppresses crack propagation by counteracting the tensile stress at tips (Fig. 14e), which can mitigate the chemo–mechanical degradation in cathode. These finding suggest that the line defects, commonly viewed as harmful, may be strategically used to enhance the operational stability of cathode materials. In an earlier study, GPA technique was performed to investigate the structural evolution of LiCoO_2_ cathode after high voltage delithiation, revealing the formation of coherent twin boundaries and anti-phase domain boundaries. This finding offers important mechanistic insights for developing high-performance cathode materials in all-solid-state battery systems (Fig. 14f-h) [85].

In certain cases, structural strain is prone to the formation of various defects, which significantly affect ion transport dynamics. By conducting in-situ investigations on the structural and stress evolution of graphite during the lithiation process, local stresses were observed originate from the uneven distribution of lithium ions upon they intercalation into the graphite (Fig. 14i-p) [159]. These stress led to deformation of the graphite structure, resulting in the formation of various defect structures, such as dislocations and microdomains. The presence of such defects can reduce the energy barrier and facilitates lithium-ion diffusion across the graphite layers, thereby allowing defect engineering to contribute to the reaction kinetics of graphite.

These examples demonstrate that the integration of atomic-level resolution TEM images with GPA methods allows for assessing the stress and strain distributions during the structural evolution of electrode materials. Some of the findings have implications for understanding the mechanisms underlying structural degradation caused by stresses and strains. The in-situ atomic-scale assessment of mechanistic stress and strain may represent a significant advancement in understanding the dynamic structural degradation in electrode nanomaterials across various electrochemical storage systems.

## Conclusion and Outlook

Taken together, significant progress has been made in the development and applications of in-situ TEM techniques in the field of electrochemical energy storage systems. These techniques encompass the investigations of chemical and structural evolution, phase transformations, stress evolution, charge distribution, and dynamic interfacial behavior. The in-situ approaches have demonstrated their importance in gaining a thorough understanding of the effects of electrochemical processes on individual battery components, as well as in elucidating the fundamental mechanisms of electrochemical reactions, degradations, and failures. The advanced real-time information provided by these techniques offers invaluable insights into the underlying mechanisms of materials degradation, thereby guiding the optimization and innovation of battery materials and ultimately contributing to the enhancement of overall performance of the energy storage devices. While notable progress has been achieved in the development of in-situ TEM equipment and characterization techniques, there are still many challenges, highlighting the need of more in-depth fundamental understanding of the interfacial structures in view of the rapid development of various new electrochemical energy storage systems and their potential applications in the global drive seeking renewable energy sources. Some of the main challenges and future research directions associated with in-situ TEM techniques for studying the electrochemical interfacial structures are summarized in the following research fronts.

(i) There are still technical challenges in applying the existing in-situ TEM sample holders for achieving high resolution in real-time observation of the lattice structure evolution of electrode materials during electrochemical reactions. The technical difficulties stem from two main aspects. First, the current sample holders used for in-situ TEM experiments are single-tilt holders, which are not conducive to finding the zone axis of the sample through rotation, thereby preventing the acquisition of clear lattice images. Therefore, advanced in-situ TEM techniques based on designed double-tilt holder should be developed. Second, atomic-resolution HAADF and annular bright field (ABF) images require acquisition in STEM mode, where the lattice structure of the materials is easily damaged by prolonged electron beam irradiation. Reducing the operating voltage of the TEM can effectively mitigate the damage of electron irradiation, but it may also lead to a certain degree of reduction in the resolution of electron images.
(ii) There are limitations in using some of the current open electrochemical cells in the in-situ TEM experiments due to ineffective battery-performance cycles. In many cases, the electrode material dynamics cannot be uncovered during battery’s operation over long-term cycling. To address this, an accelerated durability test could be adopted, which should aid elucidating the inherent fundamental science of structural transformation process over long-term cycling.
(iii) For assessing intercalation reactions of the electrode nanomaterials, the determination of the transport pathways and mechanisms of intercalated ions within the host structure is rather difficult, which poses a major challenge for understanding the fundamental principles of the reaction. Current in-situ TEM techniques, when combined with EDX or EELS, allow the observation of electrochemical reaction interfaces, but have limited capabilities to capture the movement or transport of intercalated ions within the lattice. This difficulty arises partly from the limited resolution of current transmission electron microscopes for light elements, making it challenging to capture intercalated lithium and sodium ions. To overcome this, in-situ observation under STEM mode should be developed.
(iv) While charge transfer plays an important role in electrochemical energy storage, there is a lack of techniques to directly characterize charge density at the nano or atomic scale. 4D-STEM offers high temporal resolution, enabling the mapping of charge density distribution within a sample by analyzing the phase changes that occur as the electron beam propagates through the sample. 4D-STEM combined with in-situ techniques can be utilized to simulate the dynamic evolution of interfacial charge density under cycling conditions.
(v) During in-situ TEM measurements, exposure of samples to the electron beam may have a significant impact on the experimental results. High-energy electron beams may damage sample structures, particularly in organic materials and lithium metal, leading to bond breakage, oxidation, and structural degradation. As the magnification increases, the damage caused by the electron beam becomes more severe. Therefore, a lower resolution TEM mode is typically employed to mitigate the electron beam effect on the experimental results during in-situ observation. It is particularly important to take appropriate preventive measures regarding the effects of the electron beam, especially in view of some nanomaterials being sensitive to electron beam-induced reactions [73, 74]. Within this field, increasing imaging speed to reduce radiation doses and applying voltages below the threshold for radiation damage have proven effective in mitigating electron beam damage. However, the adoption of this strategy could result in a certain degree of reduction in image resolution. The integration of cryo-TEM technology with in-situ techniques offers a promising solution to electron irradiation issues. At extremely low temperatures, the damage from electron-beam can be greatly reduced. By combining cryo-TEM with in-situ TEM techniques, it is expected to obtain more meaningful information on the formation of SEI layer and lithium dendrites at the nanoscale.
(vi) In-situ TEM can facilitate the high-resolution imaging and spectroscopic data collection, but the analysis of such data is complex and time-consuming. For instance, hundreds of SAED and HRTEM images can be collected in investigating the structural evolution of electrode materials. However, analyzing and processing each image may take several months. The use of AI-aided data analysis can effectively address this challenge. AI is powerful in analyzing visual data, such as morphological images, satellite imagery, and video footage. The application of machine learning algorithms facilitates the automatic indexing of electron diffraction patterns, alongside the classification of image or spectral features and the spatial mapping of elemental distributions. This approach greatly enhances the efficiency and accuracy of data interpretation in electron microscopy, enabling advanced structural and compositional analyses.
(vii) In-situ TEM experiments are typically conducted under applied potentials to study electrochemical reactions, but the actual overpotential that drives the electrochemical reactions is highly sensitive to the contact conditions between the electrode materials and the electrolyte. Consequently, it is challenging to quantitatively evaluate the overpotential and its relationship to the reaction kinetics derived from in-situ TEM experiments. Address this issue necessitates the development of in-situ cells with optimized three-electrode configuration, which allow direct measurement of overpotentials in in-situ electrochemical cell.

While some advancements have been achieved in the development and application of in-situ TEM technology in the research field of electrochemical energy storage systems, the fundamental understanding of the interfacial nanostructures at atomic-scale remains challenging. Wu et al. has recently demonstrated the ability to gain ensembled-averaged atomic insight into dynamic and oscillatory lattice strains of nanomaterials under fuel cell operation condition by in-situ high-energy XRD coupled pair distribution function analysis [10], which points to the potential viability of atomic-scale visualization of dynamic lattice strains of electrode materials under battery operating conditions. The development of advanced combinations of imaging, diffraction, and spectrometry techniques in in-situ TEM probing of interfacial structures is promising for enabling the visualization of morphological evolution, chemical composition changes, and phase transformation mechanisms of electrode nanomaterials during electrochemical reactions. These advances will drive the development of advanced electrochemical energy storage technologies. With continuous advancement of electron microscopy techniques, the in-situ detection capabilities are anticipated to further improve nanoscale and atomic-scale resolution and reveal new insights into fundamental correlation of the dynamic interfacial structures with the electrochemical energy storage performances in practical applications.
